# Endoplasmic Reticulum Aminopeptidase-1 Functions Regulate Key Aspects of the Innate Immune Response

**DOI:** 10.1371/journal.pone.0069539

**Published:** 2013-07-24

**Authors:** Yasser A. Aldhamen, Sergey S. Seregin, David P. W. Rastall, Charles F. Aylsworth, Yuliya Pepelyayeva, Christopher J. Busuito, Sarah Godbehere-Roosa, Sungjin Kim, Andrea Amalfitano

**Affiliations:** Department of Microbiology and Molecular Genetics, Michigan State University, East Lansing, Michigan, United States of America; McGill University, Canada

## Abstract

Endoplasmic reticulum aminopeptidase-1 (ERAP1) is a multifunctional, ubiquitously expressed enzyme whose peptide-trimming role during antigen processing for presentation by MHC I molecules is well established, however, a role for ERAP1 in modulating global innate immune responses has not been described to date. Here we demonstrate that, relative to wild type mice, mice lacking ERAP1 exhibit exaggerated innate immune responses early during pathogen recognition, as characterized by increased activation of splenic and hepatic NK and NKT cells and enhanced production of pro-inflammatory cytokines such as IL12 and MCP1. Our data also revealed that ERAP1 is playing a critical role in NK cell development and function. We observed higher frequencies of terminally matured NK cells, as well as higher frequencies of licensed NK cells (expressing the Ly49C and Ly49I receptors) in ERAP1-KO mice, results that positively correlated with an enhanced NK activation and IFNγ production by ERAP1-KO mice challenged with pro-inflammatory stimuli. Furthermore, during pathogen recognition, ERAP1 regulates IL12 production by CD11c^+^ DCs specifically, with increases in IL12 production positively correlated with an increased phagocytic activity of splenic DCs and macrophages. Collectively, our results demonstrate a previously unrecognized, more central role for the ERAP1 protein in modulating several aspects of both the development of the innate immune system, and its responses during the initial stages of pathogen recognition. Such a role may explain why ERAP1 has been implicated by GWAS in the pathogenesis of autoimmune diseases that may be precipitated by aberrant responses to pathogen encounters.

## Introduction

The antigen-processing pathway generates MHC class I binding peptides via a multitude of steps. Initially, endogenous self or foreign proteins are degraded by the cytoplasmically located proteasome, generating long stretches of peptides that contain hydrophobic C-terminal residues. These peptides are further processed by the cytosolic aminopeptidases [1], or directly transferred to the endoplasmic reticulum (ER) by the transporter associated with antigen processing (TAP) where endoplasmic reticulum aminopeptidase-1 (ERAP1) a multifunctional, IFNγ-inducible, ubiquitously expressed and soluble monomeric zinc-metallopeptidase is present. The ER-localized ERAP1 further trims peptide precursors to generate or destroy antigenic epitopes prior to loading onto MHC class I molecules [2], [3], [4]. As a result, ERAP1 can play a critical role in modulating the adaptive immune responses to both host and pathogen derived antigens. For example, ERAP1-deficient mice die from *T. gondii* infection (in contrast to WT mice), due to an inability of the ERAP1 deficient mice to process (trim) and present a immunodominant 10-mer peptide derived from *T. gondii* on their MHC I molecules [5]. The role of ERAP1 in shaping the antigenic peptide repertoire against viral infections has been best described in murine models of lymphocytic choriomeningitis virus (LCMV) infection [6]. In contrast to WT mice infected with LCMV, ERAP1-KO mice generated a dramatically different hierarchy of LCMV derived immunodominant epitopes on their MHC class I molecules. LCMV derived epitopes that do not respond in WT mice, due to degradation by ERAP1, are able to become robust, immunodominant LCMV derived epitopes in ERAP1-KO mice. Conversely, LCMV epitopes that are normally generated as a result of normal ERAP1-mediated trimming activity were not generated in ERAP1-KO mice [6]. The importance of ERAP1 in modulating immune responses to pathogens is further validated by the finding that some viruses (e.g. human cytomegalovirus (HCMV)) have evolved immune evasion strategies that specifically target ERAP1 expression, for example, by viral expression of miR-US4-1 microRNAs [7]. In mice that are not infected with a pathogen, lack of ERAP1 also significantly alters the composition of the normal MHC class I “peptidome” being presented in the animals. Adoptive transfer of syngeneic splenocytes from ERAP1-KO mice into WT mice (and vice versa) has been shown to induce potent CD8+ T cell responses to the transferred splenocytes, directly confirming that lack of ERAP1 significantly alters the composition of the endogenous MHC class I peptidome [8], [9]. At the molecular level, the complete lack of the ERAP1 protein resulted in significant increases in the length of all peptides bound to the MHC class I molecules present in cells derived from ERAP1 deficient animals [10], [11].

ERAP1 has also been reported to directly cleave, or promote the cleavage of cytokine receptors normally present on the cell surface [12]. Cell culture based experiments suggested that membrane associated ERAP1 directly binds to the IL6 receptor, and that ERAP1 catalytic activity is required for cleavage of the IL6 receptor to occur [13]. Similarly, in HUVEC cells, cleavage of the TNF receptor, TNFRI, is known to be mediated by ERAP1 [14], but again it is unclear if ERAP1 directly cleaves TNFRI, or if ERAP1 indirectly increases the activity of an unknown TNFRI sheddase [11]. A physiological role for these sheddase functions remains uncertain, as ERAP1 does not significantly alter the rate of cytokine receptor shedding in mice [12].

It has also been proposed that ERAP1 has an important role in modulating tumor susceptibility. ERAP1 is over-expressed in a number of tumor cell lines, and ERAP1-deficient tumor cells are more susceptible to NK cell mediated lysis, the latter possibly due to altered interactions of MHC class I molecules with the NK cell inhibitory receptors (e.g. Ly49C) [15], [16], [17]. These limited studies have started to shed light on the important role that ERAP1 may have in modulating the innate immune system.

In the following study, we investigated the role(s) that ERAP1 may have in modulating aspects of the innate immune responses during early stages of exposure to pro-inflammatory stimuli *in vivo*. Here, we used two well described pathogen associated stimuli that are potent stimulators of the murine innate immune response: conventional Adenovirus (Ad) vectors or recombinant *Eimeria tenella* derived antigen (rEA) [18], [19], [20]. Overall we found that several arms of the innate immune system respond abnormally (excessively) in mice lacking ERAP1 following exposure to these, pathogen derived inflammatory stimuli. These findings suggest that the ERAP1 protein may play a previously unrecognized, global role in modulation of the immune system overall, and in maturation of NK cells in particular, beyond its more well known roles in antigen processing and presentation pathways.

## Materials and Methods

### Ethics Statement

Animal handling, including injections, and plasma and tissue sample collections were performed in accordance, and with approval of the Michigan State University Institutional Animal Care and Use Committee. All procedures with recombinant Ads were performed under BSL-2, and all vector treated animals were maintained in ABSL-2 conditions. All animal procedures performed in this study were reviewed and approved by the Michigan State University EHS, IBC, and IACUC (AUF number: 03/10-030-00). Care for mice was provided in accordance with PHS and AAALAC standards ((ID number: A3955-01).

### Adenovirus Vector Construction, Production and Characterization

Adenovirus vectors (Ads) were constructed as detailed [19]. Briefly, the following Ads were utilized: Ad5-GFP and Ad5-HIV-Gag [19], [21], [22]. All Ads utilized in this study were human Ad type 5 derived replication deficient vectors (deleted for the E1 and E3 genes). Construction of, and viral propagation were each completed as described [19], [22], [23]. All viruses were found to be RCA free both by RCA PCR (E1 region amplification) and direct sequencing methods as previously described [20]. All Ads have also been tested for the presence of bacterial endotoxin as previously described [20] and were found to contain<0.15 EU per ml.

### Animal Procedures

Adult C57BL/6 WT mice were purchased from Taconic Farms (Germantown, NY). C57BL/6 ERAP1 knockout mice were a kind gift from Dr. Kenneth Rock (Professor, University of Massachusetts Medical School). Ad5 vectors were injected intravenously (via the retro-orbital sinus, total volume 200 µl, diluted in PBS) or intramuscularly (into the tibialis anterior of the right hindlimb, total volume 25 µl) into 6–8 week old male mice after performing proper anesthesia with isofluorane. A total of 1.5×10^11 ^vp per mouse was used for IV injections, 2×10^10^ vp per mouse was administered IM. Intraperitoneal (IP) injection of animals (6–8 weeks in age) consisted of 100 µl phosphate-buffered saline solution (PBS, pH 7.4) containing 100 ng rEA protein from Eimeria *tenella* as previously described [24], [25]. rEA protein purification was performed as previously described [24] with minor modifications described [25].

### Cytokine and Chemokine Analysis

A mouse-based 23-plex multiplex-based assay was used to determine the indicated cytokine/chemokine concentrations in the plasma samples collected from WT or ERAP1-KO mice at the specified time points after IV, IM, or IP treatment with Ad or rEA innate stimuli. Assay was performed according to the manufacturer’s instructions (Bio-Rad, Hercules, CA) via Luminex 100 technology (Luminex, Austin, TX) as previously described [20].

### Isolation of Lymphocytes from Spleen and Liver Tissues

Splenocytes from individual mice were harvested and processed as follows: spleen tissues were physically disrupted (by passage through a 40 µm sieve, followed by RBCs lysis by using 2 ml of ACK lysis buffer (Invitrogen, Carlsbad, CA) per homogenized spleen. Splenocytes were subsequently washed two times with RPMI medium 1640 (Invitrogen, Carlsbad, CA) supplemented with 10% FBS, 2 mM L-glutamine, 1% PSF (penicillin, streptomycin, fungizone), resuspended, and counted. Liver tissue from individual mice was minced into small pieces, incubated in complete RPMI with collagenase/DNase for 1 hour at 37°C, with vigorous vortexing every 15 minutes. Following incubation, tissue was passed through a 40 µm sieve, followed by RBC lysis and resuspension in complete RPMI media [25].

### Cell Staining and Flow Cytometry

Early activation of NK, NKT, B, T cells, dendritic cells, and macrophages in C57BL/6 WT and ERAP1-KO mice, stimulated with Ad (IM) or rEA (IP) was studied by Flow Cytometry based methods. At 6 and 12 hpi (rEA [18]) or 12 (Ad [26]) hours after injection mice were sacrificed and splenocytes and liver lymphocytes prepared. For surface antibody staining, two million cells were washed two times with cold FACS buffer, incubated for 15 minutes with purified rat anti-mouse CD16/CD32 Fcγ block (BD Biosciences, San Diego, CA), washed with FACS buffer and incubated on ice for 30 minutes with the following antibodies: CD69-PE, CD3-APC-Cy7, CD3-APC, CD3-PerCpCy5.5, CD19-PerCpCy5.5, NK1.1-PE-Cy7, NK1.1-APC, DX5-PE-Cy7, CD43-FITC, CD11b-PE, Ly49A-PE, Ly49A-AmCyan-A, Ly49C-PE, Ly49C/I-PE, Ly49G-FITC, Ly49D-FITC and CD8a-AlexaFluor700 (all 4 µg/ml, all from BD Biosciences, San Diego, CA). For dendritic cells activation, splenocytes were stained with the following antibody cocktail: CD11b-APC-Cy7, CD11c-PE-Cy7, CD86-Pacific_Blue, CD80-PE, MHCII-AlexaFluor700, and CD40-FITC (all 4 µg/ml, all from BD Biosciences, San Diego, CA).

For intracellular staining, 4 million splenocytes were plated; Brefeldin A was added to a final concentration of 1 µg/ml and incubated for 3 hours at 37°C. Following incubation, splenocytes were washed two times with FACS buffer, incubated with Fcγ block, surface stained with CD3-PerCp-Cy5.5 and NK1.1-PE-Cy7, fixed with 2% formaldehyde for 20 minutes on ice, permeabilized with 0.5% Saponin for 20 minutes at room temperature, and incubated on ice with IFNγ-APC (8 µg/ml) for 2 hours. Data were collected on BD LSR II instrument and analyzed using FlowJo software (Tree Star, San Carlos, CA, USA).

### Cytokine Assays for Evaluating NK Cells Licensing

Splenocytes were harvested and stimulated with 0.6 µg or 2 µg of PK136 (anti-NK1.1) mAbs essentially as previously described [27]. Briefly, six-well tissue culture-treated plates were coated with 0.6 µg or 2 µg purified PK136 in 1 ml PBS. A total of 10^7^ naive splenocytes were added to the washed plates and incubated at 37°C and 5% CO2 for 1 hour and then further incubated in the presence of brefeldin A (GolgiPlug, BD Biosciences) for an additional 7 hours. For IL2 and IL18 stimulations, splenocytes were stimulated with these cytokines in the presence of berfeldin A as mentioned above. IFN-γ was detected by intracellular cytokine staining and flow cytometry as described previously [28].

### Measurement of Phagocytic Activity

Phagocytosis was assessed by measuring the cellular internalization of latex beads coated with FITC-labeled rabbit IgG into cells using a phagocytosis assay kit (Cayman Chemical, Ann Arbor, MI); performed according to the manufacturer’s protocol. Briefly, total splenocytes (4×10^6^ cells/well), prepared from C57BL/6 WT or ERAP1-KO mice, uninfected or injected with Ad5-HIV-Gag for 12 hours (IM), were cultured in 24-well plates containing RPMI-1640 supplemented with 10% fetal bovine serum (FBS) and 1% penicillin–streptomycin-fungizone and latex beads. Following incubation for 24 hours at 37°C, uptake of the beads into cells was evaluated by FACS, with data collected on BD LSR II instrument and analyzed using FlowJo software.

For macrophage and DCs phagocytosis, splenocytes (4×10^6^ cells/well) derived from naïve or rEA-treated C57BL/6 WT or ERAP1-KO mice were harvested and plated in 24-well plates. Cells were then treated with latex beads for 24 hours and cultured at 37°C. After 24 hours, cells were washed twice with FACS buffer and stained with PE-Cy7 conjugated CD11b and APC-Cy7 conjugated CD11c antibodies. Data were collected using a BD LSR II instrument and analyzed using FlowJo software.

### Ad Transduction Efficiency in C57BL/6 WT and ERAP1-KO Mice

To determine the number of Ad genome copies per liver cell at 6 hours post IV injection in C57BL/6 WT or ERAP1-KO mice, liver tissues (<0.1 g) were snap frozen in liquid nitrogen, crushed to a fine powder using a mortar and pestle and total DNA was extracted as previously described [20], [29]. Ad genome copy numbers were assessed using Real-Time PCR based quantification as previously described [20], [23], [29]. To determine if Ad-GFP transduction capabilities upon IM injection into C57BL/6 WT and ERAP1-KO mice were similar, total splenocytes were prepared at 12 hpi, surface stained with CD3-APC-Cy7 and NK1.1-PE-Cy7 (8 µg/ml) and evaluated by FACS, with data collected using an LSR II instrument and analyzed using FlowJo software. Total lymphocytes or NK cells (CD3**^−^**, NK1.1^+^) were analyzed for GFP expression (FITC channel).

### Mouse Dendritic Cells Isolation and mIL12p70 ELISA

An *in vitro* bioassay originally developed to monitor rEA activity during its purification and production, as well as provide some insight into the mechanism of action of rEA, was previously described [18], [24]. The assay is based upon monitoring the release of mIL12(p70) from mouse DCs as an indicator of rEA-induced DC activation. Briefly, mouse DCs (CD11c+ splenocytes) were isolated from C57BL/6 WT and ERAP1-KO mice using the magnetic assisted cell separation (MACS) system (Miltenyi Biotech, Auburn, CA) utilizing the established protocols from the manufacturer and previously described [18], [24]. Isolated CD11c+ cells (at least 85% pure, data not shown) were seeded at a density of 0.5×10^5^ cells per well in 96-well plates in 200 µl/well complete medium. Mouse DCs were stimulated with rEA (10**^−^** ^6^–10^1^ ng/ml) overnight (∼16 hours) at 37°C in 5% CO2 and 95% ambient air. Following incubation, culture medium was analyzed for mouse IL12p70 levels using an ELISA kit and following its enclosed instructions (R&D Systems, Minneapolis, MN).

### Quantitative RT-PCR

To study the activation of transcription of pro-inflammatory genes upon triggering the innate immune system by IV injection of 1.5×10^11^ vp/mouse Ad5-HIV-GAG into C57BL/6 or ERAP1-KO mice, animals were sacrificed at 6 hpi and RNA was harvested using TRIzol reagent (Invitrogen, Carlsbad, CA, USA) per the manufacturer’s protocol. Following RNA isolation, reverse transcription was performed on 1 µg of total RNA using SuperScript III (Invitrogen, Grand Island, NY) reverse transcriptase (RT) and random hexamers/oligo (dT) primers per manufacturer’s protocol. RT reactions were diluted to a total volume of 60 µl, and 2 µl was used as the template in the subsequent PCR reactions. Primers were designed using Primer Bank web-based software (http://pga.mgh.harvard.edu/primerbank/); complete list of primers used is available upon request. Quantitative PCR (qPCR) was carried out on an ABI 7900HT Fast Real-Time PCR System as detailed [19], [20].

### Statistical Analysis

For every experiment, pilot trials were performed with n = 3 per group. This allowed us to determine effect size and sample variance so that Power Analysis could be performed to correctly determine the number of subjects per group required to achieve a statistical Power >0.8 at the 95% confidence level. Statistically significant differences in innate immune responses were determined using One Way ANOVA with a Student-Newman-Keuls post-hoc test (p value<0.05) or by using two-tailed homoscedastic Student’s t-tests. Graphs in this paper are presented as Mean of the average ± SEM, unless otherwise specified. GraphPad Prism software was utilized for statistical analysis.

## Results

### In Response to Several Inflammatory Stimuli, Mice Lacking ERAP1 Exhibit Significantly Elevated Levels of NK and NKT Cell Activation

The goal of this study was to investigate the hypothesis that ERAP1 plays an important role in modulation of the innate immune system, *in vivo.* To examine this, two distinct and previously well-described stimulators of the innate immune system of the mouse were utilized: recombinant, *Eimeria tenella* derived antigen (rEA) [24] or Adenovirus (Ad) vectors [18], [20], [30].

**Figure 1:**
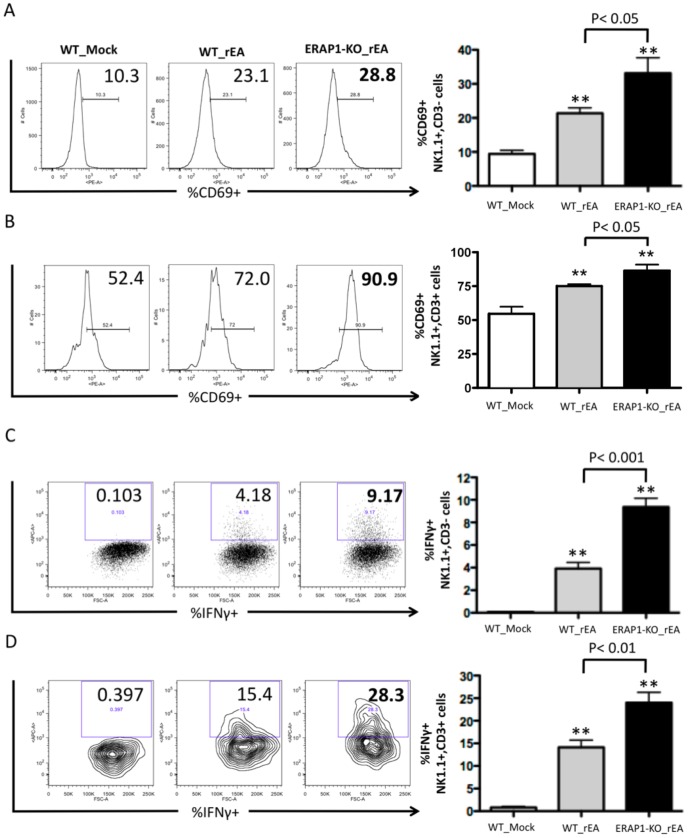
Mice lacking ERAP1 exhibit drastically increased activation of NK and NKT cells in the liver in response to rEA stimuli. C57BL/6 WT and ERAP1-KO mice were either mock (PBS) injected or intraperitoneally injected with 100 ng/mouse of rEA protein. Liver lymphocytes were prepared at 6 hpi, processed, stained for expression of surface markers (intracellular staining was performed for IFNγ), and analyzed by FACS as described in Materials and Methods. (**A, B**) CD69 activation and (**C, D**) IFNγ release by NK cells (**A, C**) and NKT cells (**B, D**) are shown. Bars represent mean ± SEM. Representative plots are shown. Statistical analysis was completed using a one-way ANOVA with a Student-Newman-Keuls post-hoc test. n = 4 for all groups of mice. *, ** - indicate values, statistically different from those in mock-injected mice, p<0.05, p<0.001, respectively.

**Figure 2:**
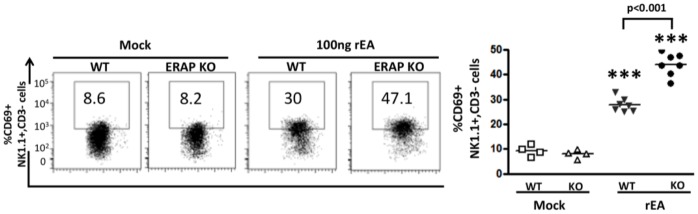
Mice lacking ERAP1 exhibit increased activation of NK cells in the spleen in response to rEA stimuli. C57BL/6 WT and ERAP1-KO mice were either mock (PBS) injected or intraperitoneally injected with 100 ng/mouse of rEA protein. Splenocytes were prepared at 12 hpi, processed, stained for expression of surface markers and analyzed by FACS as described in Materials and Methods. CD69 activation NK cells is shown, Bars represent mean ± SEM. Representative plots are shown. Statistical analysis was completed using a one-way ANOVA with a Student-Newman-Keuls post-hoc test. n = 4–7 for all groups of mice. *** - indicate values, statistically different from those in mock-injected mice, p<0.0001.

We have previously reported that injections of rEA or conventional Ad5 vectors results in significant activations of NK and NKT cells both in the liver and in the spleens of WT mice [18], [25]. In this study, Intraperitoneal (IP) injection of rEA protein into ERAP1-KO mice resulted in increased activation of hepatic NK cells (CD3**^−^**NK1.1^+^) and CD3^+^NK1.1^+^ NKT-cells (p<0.05), as compared to similar injections of rEA into WT mice (Figure 1A and 1B). Moreover, in response to rEA, ERAP1-KO mice had significantly (p<0.001) increased levels of IFNγ secretion by both hepatic NK and NKT cells, as compared to WT mice (Figure 1C and 1D). Specifically, in rEA treated ERAP1-KO mice 8% of hepatic NK cells produced IFNγ, as compared to ∼4% of hepatic NK cells producing IFNγ in identically treated WT mice. Similarly, ∼25% of the hepatic NKT cells in ERAP1-KO mice secreted high levels of IFNγ in response to rEA, as compared to only ∼15% of hepatic NKT cells in rEA treated WT mice (p<0.001) (Figure 1C and 1D). In the spleen at 6 hours post injection (hpi), both NK and NKT cells isolated from WT and ERAP1-KO mice had similar CD69 activation levels in response to rEA (Figure S1A). IFNγ secretion by splenic NK cells was dramatically enhanced in response to rEA stimulation in both WT and ERAP1-KO mice (from ∼0.4 to ∼20%), but with no significant differences between rEA treated WT and ERAP1-KO mice (Figure S1B). However, greater numbers of splenic NKT cells were able to secrete increased levels of IFNγ in rEA-treated ERAP1-KO mice (∼16%) as compared to rEA treated WT mice (∼10%) (p<0.05) (Figure S1B). We also evaluated the activation of NK and NKT cells at 12 hpi following rEA stimulation and observed significantly (p<0.001) increased percentages of CD69-expressing NK cells in the spleens of ERAP1-KO mice, as compared to identically injected WT mice (Figure 2). Activation levels of NKT cells also trended to increase in cells derived from ERAP1-KO mice, however not to statistically significant levels (Fig. S1C).

To further verify that ERAP1 is involved in mediating activation of NK and/or NKT cells in response to inflammatory molecules or pathogens, we intramuscularly (IM) injected WT and ERAP1-KO mice with conventional Ad5 vectors. At 12 hpi, Ad-injected ERAP1-KO mice had significant increases in the frequency of IFNγ producing NK cells (10% in ERAP1-KO as compared to 5% in Ad infected WT mice; p<0.05) and in the number of activated (CD69 expressing) NKT cells (60% in ERAP1-KO, 50% in WT to, p<0.01) (Figure S2A), but there were no significant differences in the activation of splenic NK cells in ERAP1-KO mice as compared to Ad-injected WT mice (Figure S2B).

**Figure 3:**
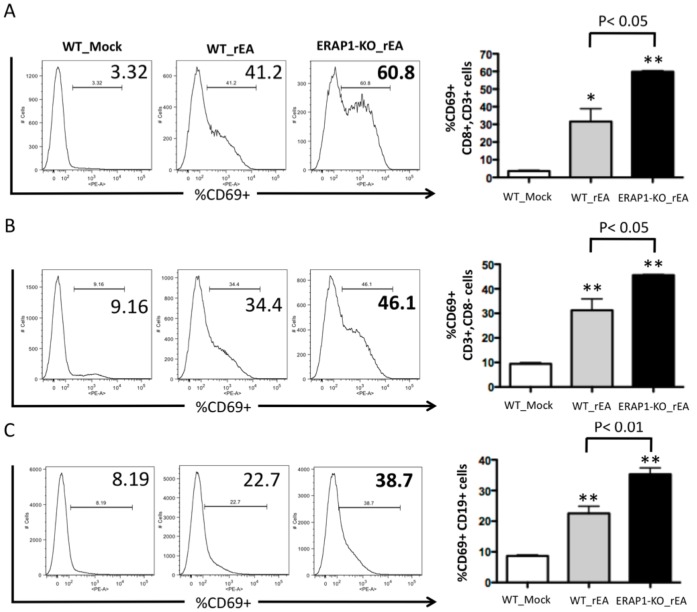
Mice lacking ERAP1 exhibit dramatically enhanced activation of B cells, CD8^+^ and CD8^−^ T cells in response to rEA stimuli. C57BL/6 WT and ERAP1-KO mice were either mock (PBS) injected or intraperitoneally injected with 100 ng/mouse of rEA protein. Splenocytes were harvested at 6 hpi, processed, stained for expression of surface markers, and analyzed by FACS as described in Materials and Methods. CD69 activation of (**A**) CD8^+^ CD3^+^ T cells, (**B**) CD8**^−^** CD3^+^ T cells, and (**C**) B cells are shown. Bars represent mean ± SEM. Representative plots are shown. Statistical analysis was completed using a one-way ANOVA with a Student-Newman-Keuls post-hoc test. n = 4 for all groups of mice. *, ** - indicate values, statistically different from those in mock-injected mice, p<0.05, p<0.001, respectively.

Overall levels of activated (CD69^+^) B and T cells were also found to be significantly different between rEA-treated WT and ERAP1-KO mice. Specifically, we detected significantly (p<0.05) higher levels of CD19^+^CD69^+^ B cells in rEA-treated ERAP1-KO mice (∼55%), as compared to rEA-treated WT mice (∼30%), (Figure 3A). Moreover, we detected significantly (p<0.05) higher levels of CD69^+^CD3^+^CD8**^−^** T cells (Figure 3B) and CD69^+^CD3^+^CD8^+^ T cells (p<0.01) (Figure 3C) in rEA-treated ERAP1-KO mice as compared to rEA-treated WT mice. However, levels of CD69^+^ B cells, CD3^+^CD8^+^ and CD3^+^CD8**^−^** T cells were not significantly different between WT and ERAP1-KO mice in response to Ad injections (Figure S3A-C).

#### ERAP1 is involved in the terminal steps of NK cell maturation

**Figure 4:**
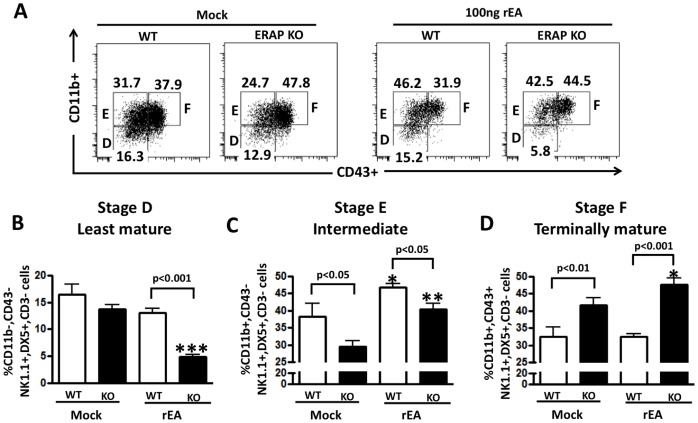
ERAP1-KO mice have significantly enhanced NK cell maturation (larger number of stage F mNK cells) as compared to WT mice. WT C57BL/6 and ERAP1-KO mice (n = 4 for mock groups and n = 7 for rEA injected mice) were either mock (PBS) injected or intraperitoneally injected with 100 ng/mouse of rEA protein. Splenocytes were harvested at 12 hpi, processed, stained for expression of NK cells maturation surface markers, and FACS analysis was performed as described in materials and methods. (**A**) Representative figures of the frequency of CD11b and CD43 expressing-mNK cells gated from CD3−, NK1.1+, and DX5+ cells in spleens of mock or rEA treated ERAP1-KO versus wild type mice. (**B**) Percentages of stage D least mature (CD11b-, CD43-) NK cells (**C**) Percentages of stage E intermediate (CD11b+, CD43-) mature NK cells. (**D**) Percentages of stage F terminally mature (CD11b+, CD43+) NK cells. Data was collected in LSR-II and analyzed by FlowJo software. The bars represent mean ± SEM. Statistical analysis was completed using a one-way ANOVA with a Student-Newman-Keuls post-hoc test. p<0.05 was deemed a statistically significant difference. *, **, *** - indicate values, statistically different from those in mock-injected mice, p<0.05, p<0.01, p<0.001, respectively.

To investigate if ERAP1 is involved in NK cell maturational processes that may correlate with NK responses to inflammatory stimuli, we analyzed mature NK cells (defined as CD3−, NK1.1+, DX5+ cells) and their developmental stage (least mature (stage D; CD11b-, CD43-), intermediate (stage E; CD11b+ (Mac-1), CD43-), and terminally mature (stage F; CD11b+, CD43+)) in mock treated, or rEA treated WT or ERAP1-KO mice [31], [32], [33]. We observed the presence of similar percentages of matured NK cells (mNK: CD3−, NK1.1+, DX5+) in the splenocytes of both WT and ERAP1-KO, both in the absence of, or after treatment with rEA (Figure S4). Interestingly, when we evaluated three well characterized maturational stages of these mNK cells, our data revealed that the preponderance of mNK cells derived from mock treated WT mice were in Stages E and F (Figure 4). Mock treated ERAP1-KO mice also had the preponderance of mNK cells in Stage E and F, however, mock treated ERAP1-KO mice had significantly (p<0.01) higher levels of Stage F mNK cells, and significantly (p<0.05) lower levels of Stage E mNK cells in their splenocytes, as compared to splenocytes derived from mock treated WT mice (Figure 4).

The rEA treatment of WT mice resulted in a significant (p<0.05) increase in the numbers of Stage E mNKs. This result was paralleled by slight decreases in Stage D mNKs, and no change in the numbers of Stage F mNKs in the rEA treated WT mice. rEA treatment of ERAP1-KO mice also resulted in significantly (p<0.01) increased numbers of Stage E mNKs as compared to mock treated ERAP1-KO mice, paralleling results noted in rEA treated WT mice. However, the overall net increase in numbers of Stage E mNKs was lower than that noted in WT mice (p<0.05), a result possibly due to the relatively decreased numbers of Stage E mNKs that are present in mock treated ERAP1-KO mice (as compared to WT mice). More importantly, rEA treatment of ERAP1-KO mice resulted in significant (p<0.05) increases in the numbers of Stage F mNK cells, as compared to mock treated ERAP1-KO mice. This is important, as WT mice did not demonstrate any evidence of induction of Stage F mNKs in response to rEA treatment.

This maturational shift, which promoted the development of Stage F mNKs in rEA treated ERAP1-KO mice was also supported by the observation that rEA treatment of ERAP1-KO mice resulted in significant decreases in the numbers of Stage D mNKs, in comparison to both rEA treated WT mice (p<0.001) as well as to mock treated ERAP1-KO mice (p<0.001). Together, the data suggests that in addition to differences between WT and ERAP1-KO mice in regard to the maturational stages of their existing mNK populations, ERAP1-KO mice also generate enhanced responsiveness to inflammatory stimuli that for example, resulted in large inductions of Stage F mNKs, inductions that do not occur in similarly treated WT mice.

#### ERAP1-KO mice have altered numbers of licensed NK cells

**Figure 5:**
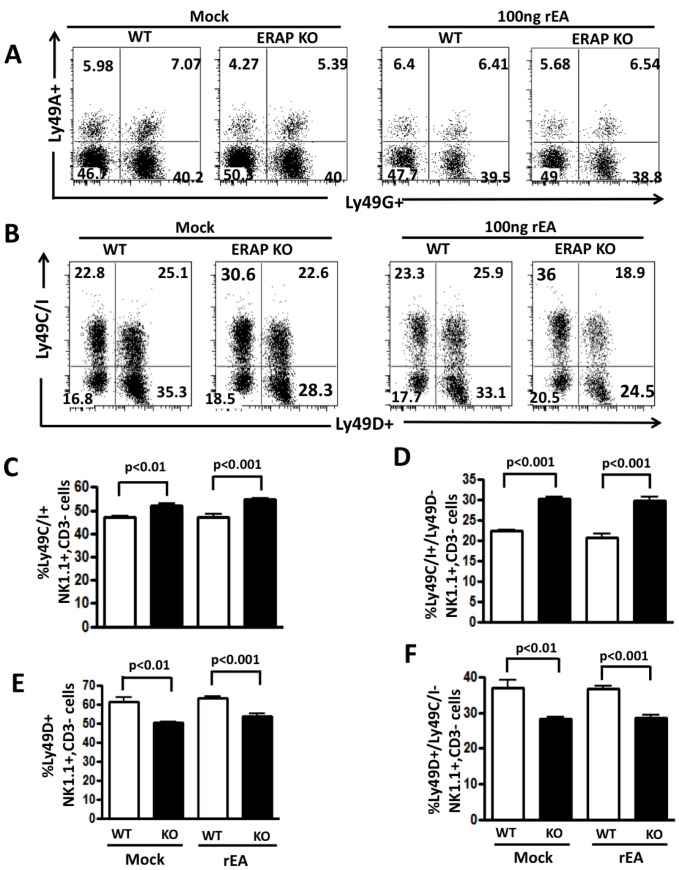
Increased number of licensed NK cells in ERAP1-KO mice. WT C57BL/6 and ERAP1-KO mice (n = 4 for mock and n = 7 for rEA injected groups of mice) were either mock (PBS) injected or intraperitoneally injected with 100 ng/mouse of rEA protein. Splenocytes were harvested at 12 hpi, processed, stained for expression of Ly49 receptors on NK cells, and FACS analysis was performed as described in materials and methods. (**A**) Representative figures of percentages of Ly49A and Ly49G expressing NK cells (CD3−, NK1.1+). (**B**) Representative figures of percentages of Ly49C, Ly49I, and Ly49D expressing NK cells. (**C**) Frequency of Ly49C/I expressing NK cells. (**D**) Frequency of Ly49D expressing NK cells. (**E**) Frequency of Ly49C/I positive and Ly49D negative NK cells. (**F**) Frequency of Ly49D positive and Ly49C/I negative NK cells. The bars represent mean ± SEM. Data was collected in LSR-II and analyzed by FlowJo software. Statistical analysis was completed using a one-way ANOVA with a Student-Newman-Keuls post-hoc test. p<0.05 was deemed a statistically significant difference.

We next determined if ERAP1 is involved in NK cell licensing. Licensing status of NK cells is typically determined by analyzing the Ly49 receptors present on them. There are four Ly49 receptors known to be expressed in C57BL/6 (H-2b) mice: Ly49A, Ly49G, Ly49C, and Ly49I [33], [34], [35]. Licensed NK cells express Ly49C and/or Ly49I, which efficiently bind to H-2Kb, whereas non- licensed NK cells only express Ly49G or Ly49A, which do not efficiently bind to H-2b [35]. NK cells can be further evaluated as to their expression of Ly49D, an activating receptor, relative to their licensing status. While we noted similar numbers of total Ly49A and Ly49G positive NK cells derived from WT and ERAP1-KO mice (Figure 5A), we noted statistically significant increases in the numbers of total Ly49C and Ly49I positive NK cells, (Fig. 5B–C and Figure S6), as well as Ly49C and Ly49I positive, and Ly49D negative NK cells (Fig. 5D) in ERAP-KO mice. In contrast, we noted that total Ly49D positive NK cells (Fig. 5E), as well as Ly49D positive, Ly49C and Ly49I negative cells (Fig. 5F) were statistically lower in ERAP-1 KO mice, as compared to WT mice. rEA treatment did not change these differences between WT and ERAP1-KO mice (Figures 5B–F). We also evaluated the production of IFNγ by licensed NK cells. NK cells derived from mock injected WT or ERAP1-KO mice were stimulated with two concentrations of anti-NK1.1 antibodies or a mixture of IL2 and IL18 cytokines and the production of IFNγ by Ly49C+ NK cells was evaluated by flow cytometry. Potent production of IFNγ was observed in NK cells derived from both WT and ERAP1-KO, however, no statistically significant differences were observed (Figure S5). Β2m-KO mice were also utilized in these experiments and consistent with previously published results, NK cells from Β2m-KO are not licensed and have defective IFNγ production (Figure S5). We also evaluated the expression of several Ly49 receptors on NKT cells, however, no significant differences were observed between WT and ERAP1-KO mice (data not shown).

### ERAP1 Modulates Production of Pro-inflammatory Cytokines and Chemokines

**Figure 6:**
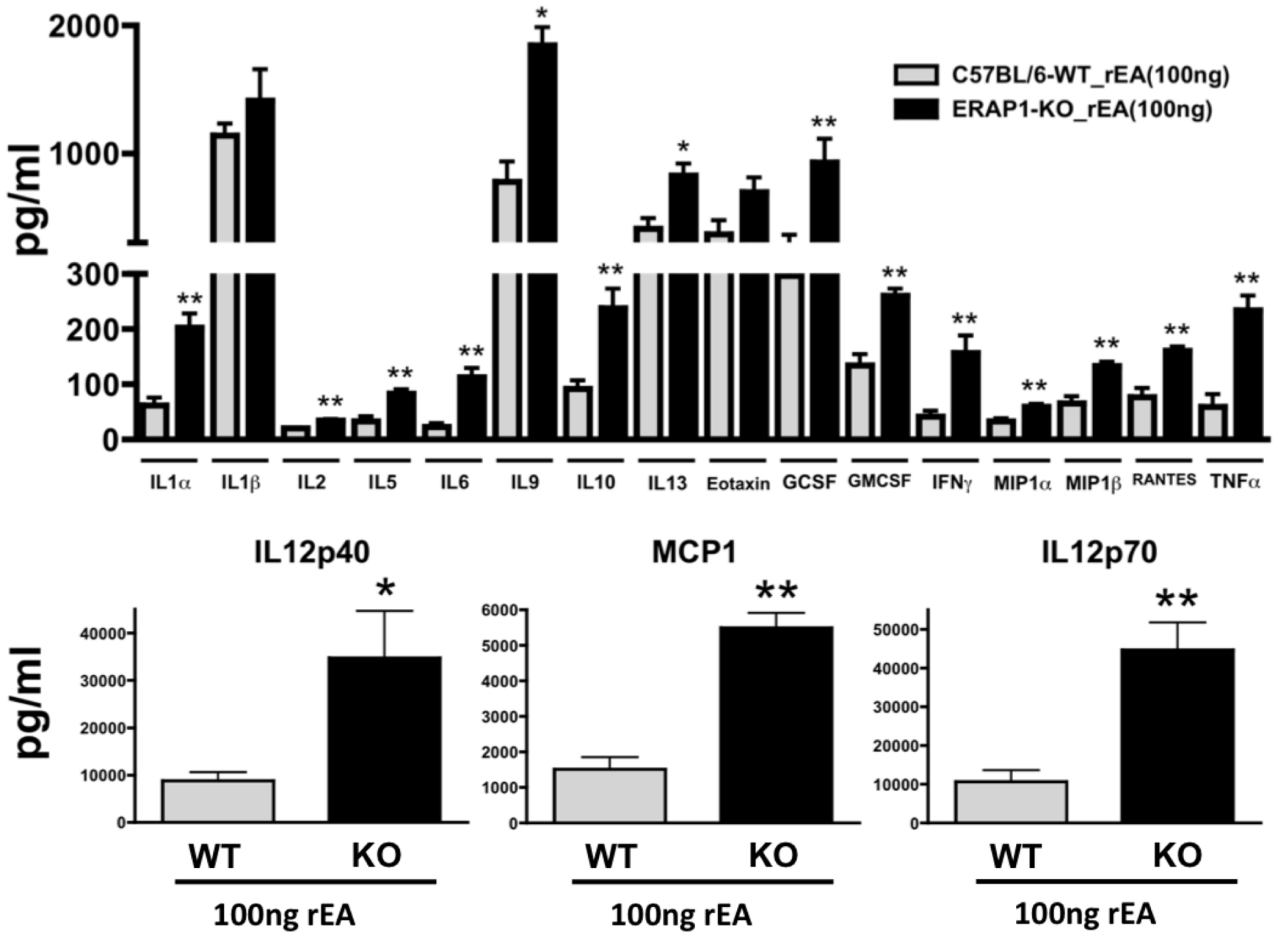
ERAP1-KO mice exhibit dramatically enhanced capabilities to respond to rEA and release pro-inflammatory cytokines. C57BL/6 WT and ERAP1-KO mice were either mock (PBS) injected or intraperitoneally injected with 100 ng/mouse of rEA protein. n = 4 for all groups of mice. Plasma samples were collected at 6 hpi and were analyzed using a multiplexed bead array based quantitative system. Bars represent mean ± SEM. Statistical analysis was completed using two-tailed homoscedastic Student’s t-tests; *, ** - indicate values, statistically different between WT_rEA and ERAP1-KO_rEA groups, p<0.05, p<0.001, respectively.

We next assessed reactive cytokine and chemokine responses of ERAP1-KO mice as compared to WT mice. We first confirmed that baseline cytokine and chemokine levels were not significantly different between WT mock and ERAP1-KO mock mice (data not shown). We recently demonstrated that, administration of the TLR11/12 agonist rEA induces systemic cytokine and chemokine releases in WT mice [18]. In this study we not only again confirmed the pro-inflammatory cytokine and chemokine inductions by rEA in C57BL/6 WT mice, but we also found that ERAP1-KO mice exhibited dramatically higher production levels of several pro-inflammatory cytokines and chemokines in response to exposure to rEA, with 3–4 fold increased levels of IL12p40, IL12p70 and MCP1, and significantly increased production levels of IL1α, IL2, IL5, IL6, IL9, IL10, IL13, GCSF, GMCSF, IFNγ, MIP1α, MIP1β, RANTES and TNFα as compared to WT mice (Figure 6). Not all of the cytokines induced by rEA were modulated by ERAP1 such as IL1β and EOTAXIN.

### Mice Lacking ERAP1 Demonstrate Exaggerated Gene Transcription Responses to Inflammatory Stimuli

We have previously confirmed that Ad injections result in global activation of a number of pro-inflammatory genes, involving multiple signaling pathways in WT mice, as detailed [19], [36]. To investigate the potential role of ERAP1 in modulation of major gene networks, and cytokine/chemokine signaling pathways, we intravenously injected conventional Ad vectors into WT and ERAP1-KO mice and then analyzed the activation of several gene networks in liver tissues of the treated animals shortly after vector administration (Tables 1–2). We initially found that baseline transcription levels of IL15 and TNFα were significantly higher in ERAP1-KO mice as compared to WT mice. In contrast, baseline MCP1 levels were significantly reduced in ERAP1-KO mice as compared to WT mice (Table 2). We found that Ad-injected ERAP1-KO mice exhibit an exaggerated activation of innate immune response genes, as compared to Ad-injected WT mice (Tables 1–2). We found that transcription of specific pattern recognition receptors were increased in Ad treated ERAP1-KO mice as compared to WT mice. Specifically, TLR6 was induced 2-fold in ERAP1-KO mice, and TLR2 and TLR3 were induced 1.3 fold, while the TLR4 and TLR9 genes were induced to similar levels after Ad treatment in both strains of mice. The TLR adaptor protein MyD88 (but not TRIF) was induced to significantly higher levels in Ad-treated ERAP1-KO mice as compared to Ad-treated WT mice. Similar patterns of specificity were noted in the expression levels of the NOD-like receptors (NLRs) genes, with NOD2 being induced 1.5 fold in Ad treated ERAP1-KO mice, while NOD1 was induced to similar levels in both strains of Ad treated mice. We also found that Ad treatment induced the expression of RIG1 and elements of the JAK/STAT signaling pathway (Jak1, STAT1, SOCS1, SOCS3) were found to be activated to similar levels in Ad-injected WT and ERAP1-KO mice (Table 1). IRF7 gene expression was higher in Ad treated ERAP1-KO mice, as compared to WT mice, but IRF3 was induced similarly in both strains of Ad treated mice. When exposed to Ads, ERAP1-KO mice also had significantly higher expression levels of several genes encoding pro-inflammatory cytokines, including: IFNβ (2 fold increase), IL1β (1.7 fold increase), IL6 (2 fold increase), IL12p40 (1.6 fold increase), and TNFα (2 fold increase). In addition, the expression of several type I IFN responsive genes were also upregulated to significantly higher levels in Ad-treated ERAP1-KO mice as compared to Ad-treated WT mice: ADAR (1.6 fold increase), CXCL9 (4 fold increase), Oas1A (2 fold increase). Several genes encoding endothelial cell activation markers were also induced to significantly higher levels in Ad treated ERAP1-KO mice as compared to WT mice (Table 2). These data suggest that ERAP1 is directly involved in regulating several innate immune pathways during inflammatory conditions and might function as a critical regulator of these signaling pathways.

**Table 1:** Signaling pathways.

	WT_Mock	ERAP1-KO_Mock	WT_Ad5-HIV-gag	ERAP1-KO_Ad5-HIV-gag
DAI	1.0±0.1	1.6±0.7	87.2±15.6^a^	106.4±36.6^a^
FynT	1.0±0.1	1.0±0.3	0.5±0.1	0.7±0.3
IRF3	1.0±0.3	1.0±0.3	1.0±0.3	1.4±0.5
**IRF7**	1.0±0.2	1.1±0.1	15.1±2.5^a^	**23.1±7.3^ab^**
IRF8	1.0±0.05	1.0±0.6	3.2±0.9^a^	4.8±2.2^a^
Jak1	1.0±0.1	0.8±0.1	1.2±0.2	1.2±0.7
**MyD88**	1.0±0.2	0.9±0.1	6.3±0.3^a^	**8.5±2.7^ab^**
NFkB	1.0±0.1	0.9±0.1	1.8±0.7	2.3±0.7^a^
NOD1	1.0±0.2	0.9±0.1	2.7±0.5^a^	3.1±1.0^a^
**NOD2**	1.0±0.03	1.3±0.7	3.8±1.0^a^	**5.6±2.2^ab^**
RIG1	1.0±0.2	1.5±0.3	13.2±4.1^a^	17.3±5.0^a^
SLAM	1.1±0.5	1.7±0.8	1.8±0.4	1.5±0.2
SOCS1	1.2±0.8	1.1±0.7	209.6±72.9^a^	217.7±95.1^a^
SOCS3	1.0±0.2	0.4±0.1	4.9±1.0^a^	4.4±1.4^a^
STAT1	1.0±0.1	1.0±0.2	17.1±4.1^a^	22.0±8.3^a^
TBK1	1.0±0.1	0.9±0.3	5.2±0.8^a^	5.9±2.0^a^
**TLR2**	1.0±0.1	1.5±0.4	128.1±50.0^a^	**163.5±63.7^ab^**
**TLR3**	1.0±0.2	0.8±0.4	27.8±12.4^a^	**37.8±17.9^ab^**
TLR4	1.0±0.2	1.2±0.3	1.4±0.3	1.8±0.6
**TLR6**	1.0±0.2	1.2±0.4	5.2±1.8^a^	**10.6±3.7^ab^**
TLR9	1.0±0.4	1.8±0.8	8.2±2.7^a^	6.3±2.5^a^
TRAF6	1.0±0.2	0.8±0.2	1.0±0.1	1.0±0.6
TRIF	1.0±0.3	1.0±0.3	1.7±0.4^a^	2.0±0.8^a^

Ad5-HIV-gag induced gene expression in livers of C57BL/6 mice (fold over WT_Mock, 6 hpi). The numbers represent Mean ± SD. Statistical analysis was completed using One Way ANOVA with a Student-Newman-Keuls post-hoc test, p<0.05 was deemed a statistically significant difference. n = 4 for all Mock injected groups, n = 6 for all Ad5-HIV-gag injected groups.

a Significant differences as compared to WT_Mock;

b significant differences in transcriptional activation in ERAP1-KO_Ad5-HIV-gag group as compared to WT_Ad5-HIV-gag group (also indicated by boldface font).

**Table 2:** Innate Immune genes.

	WT_Mock	ERAP1-KO_Mock	WT_Ad5-HIV-gag	ERAP1-KO_Ad5-HIV-gag
**ADAR**	1.0±0.3	1.1±0.3	10.4±2.1^a^	**16.3±6.0^ab^**
**CXCL9**	1.0±0.3	1.7±0.7	13.4±4.6^a^	**50.9±19.1^ab^**
**E-selectin**	1.0±0.3	1.3±0.7	4.8±1.6	**8.8±4.2^ab^**
**ICAM1**	1.0±0.3	1.3±0.4	5.4±0.9^a^	**9.6±3.6^ab^**
IFNα	1.0±0.2	1.4±0.5	1.8±0.8	2.0±1.7
**IFNβ**	1.0±0.2	1.3±0.6	2.8±0.7	**5.8±2.9^ab^**
IL1α	1.0±0.2	1.1±0.5	0.8±0.2	0.9±0.4
**IL1β**	1.1±0.7	1.4±0.5	2.5±0.8	**4.2±1.1^ab^**
**IL6**	1.0±0.2	1.5±0.6	2.2±0.5	**4.0±2.0^ab^**
**IL12p40**	1.1±0.7	2.9±1.8	33.8±11.2^a^	**53.6±20.4^ab^**
IL15	1.0±0.1	**2.6±1.0** *	2.6±0.7^a^	3.9±1.3^a^
IL18	1.0±0.1	1.0±0.2	0.9±0.1	1.2±0.4
IP10 (CXCL10)	1.0±0.5	1.6±0.9	945.3±310.0^a^	809.5±277.2^a^
MCP1	1.0±0.3	**0.2±0.1** *	3.2±1.2^a^	3.6±1.2^a^
**OAS1a**	1.0±0.2	1.1±0.3	16.1±7.3^a^	**34.2±14.5^ab^**
TGFβ	1.0±0.3	1.3±0.4	1.3±0.3	1.7±0.5
**TNFα**	1.0±0.3	**3.0±1.5** *	17.6±4.2^a^	**32.4±12.0^ab^**
Viperin	1.0±0.5	0.8±0.3	604.4±108.1^a^	530.1±247.1^a^

Ad5-gag induced gene expression in livers of C57BL/6 mice (fold over WT_Mock, 6 hpi). The numbers represent Mean ± SD. Statistical analysis was completed using One Way ANOVA with a Student-Newman-Keuls post-hoc test, p<0.05 was deemed a statistically significant difference. n = 4 for all Mock injected groups, n = 6 for all Ad5-HIV-gag injected groups.

a Significant differences as compared to WT_Mock;

b significant differences in transcriptional activation in ERAP1-KO_Ad5-HIV-gag group as compared to WT_Ad5-HIV-gag group (also indicated by boldface font).

* denotes significant differences between WT_Mock and ERAP1-KO_Mock (baseline levels).

As an important control, we confirmed that lack of ERAP1 protein does not affect the ability of Ad vectors to infect or transduce the liver (Figure S7A) or spleen (Figure S7B) tissues, or specific cell types within those tissues, such as splenic NK cells (Figure S7C).

### Dendritic Cells and Macrophages that Lack ERAP1 Exhibit Enhanced Phagocytosis

**Figure 7:**
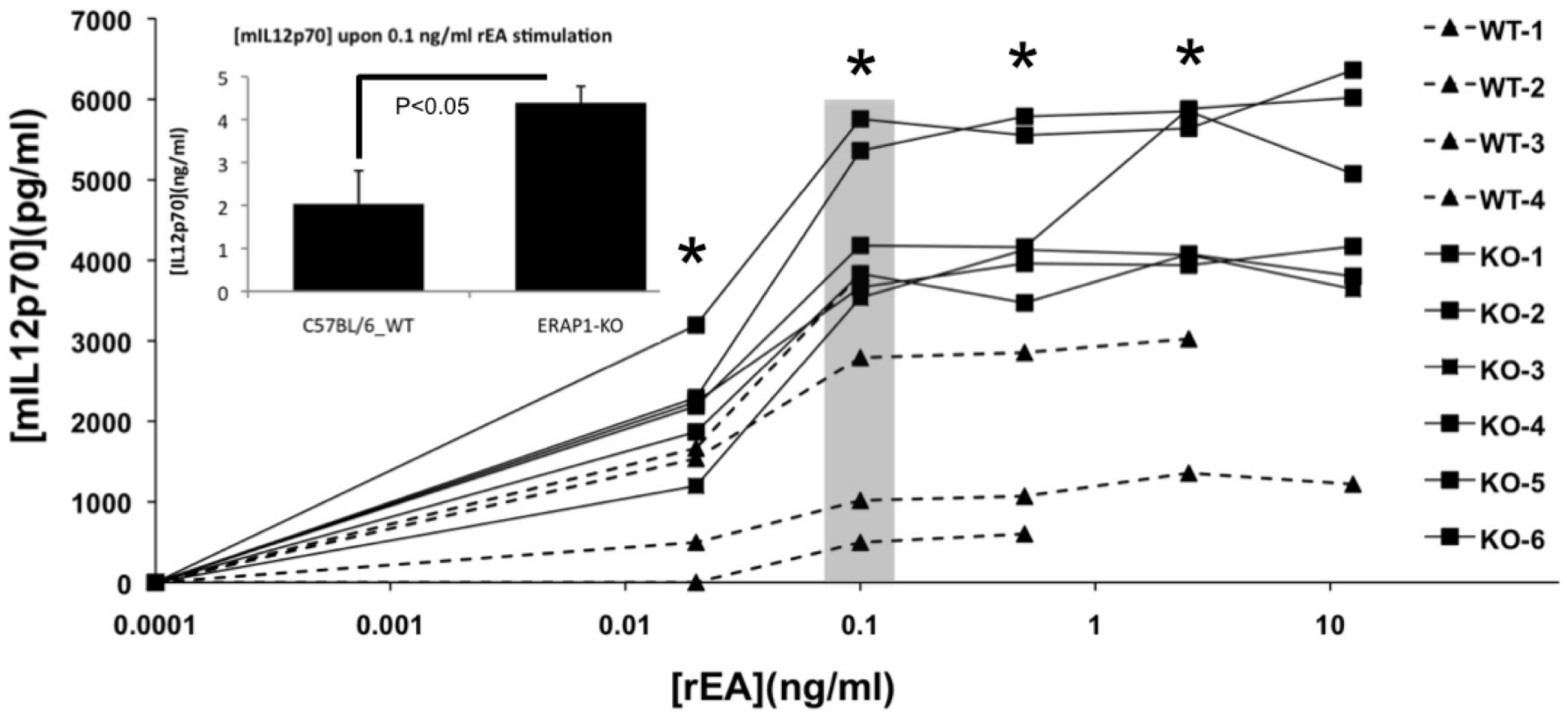
Dendritic cells derived from ERAP1-KO mice exhibit dramatically enhanced IL12p70 release when stimulated with TLR agonist rEA. CD11c+ dendritic cells were isolated from C57BL/6 WT (n = 4) or ERAP1-KO mice (n = 6) and *in vitro* stimulated with specified concentrations of rEA protein. Cultured media was used to perform an IL12p70 ELISA as described in Materials and Methods. The bars represent Mean ± SEM. 0.1 ng/ml of rEA is an optimal stimulation dose. Statistical analysis was completed using two-tailed homoscedastic Student’s t-tests; *, - indicate values, statistically different between WT_rEA and ERAP1-KO_rEA groups, p<0.05. Representative of three independent experiments is shown.

Dendritic cells (DCs) and macrophages are the main physiological producers of IL12 in response to invading pathogens [37]. Since rEA stimulation triggers significantly increased production of IL12p40 and IL12p70 in ERAP1-KO mice, we confirmed that CD11c^+^ DCs, isolated from ERAP1-KO mice have a higher capacity to respond to rEA stimulation and produce IL12 in tissue culture, as compared to similar treatments of DCs isolated from WT mice (Figure 7).

**Figure 8:**
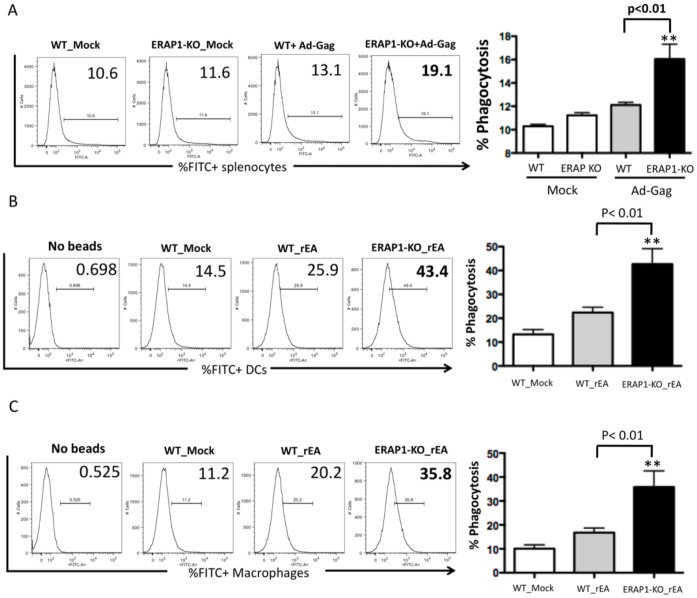
Dendritic cells and macrophages derived from ERAP1-KO mice exhibit dramatically enhanced phagocytosis capabilities in response to innate stimuli. (**A**) C57BL/6 WT and ERAP1-KO mice were either mock (PBS) injected or intramuscularly injected with 2×10^10^ vp/mouse of Ad-HIV-Gag. Splenocytes were harvested at 12 hpi, processed, incubated with FITC^+^ latex beads and analyzed by FACS as described in Materials and Methods. (**B**) C57BL/6 WT and ERAP1-KO mice were either mock (PBS) injected or intraperitoneally injected with 100 ng/mouse of rEA protein. Splenocytes were prepared at 6 hpi, processed, incubated with FITC^+^ latex beads and stained for expression of surface markers, and analyzed by FACS as described in Materials and Methods. Phagocytosis abilities of macrophages and (**C**) Dendritic cells are shown. Bars represent mean ± SEM. Representative plots are shown. Statistical analysis was completed using a one-way ANOVA with a Student-Newman-Keuls post-hoc test. n = 4 for all groups of mice. ** - indicate values, statistically different from those in mock-injected mice, p<0.001.

Efficient phagocytosis also results in IL12 production [38], [39]. Splenocytes derived from Ad treated ERAP1-KO mice had significantly (p<0.01) increased levels of phagocytic activity, as compared to Ad-treated WT mice (Figure 8A). rEA injection into ERAP1-KO mice also resulted in their DCs having significantly enhanced levels of phagocytosis (p<0.01) as compared to DCs derived from rEA treated WT mice (Figure 8B). Similarly, enhanced levels of phagocytosis (p<0.01) were also noted in macrophages derived from rEA-treated ERAP1-KO mice as compared to macrophages derived from rEA-treated WT mice (Figure 8C). We conclude that ERAP1 protein is capable of modulating phagocytic activity, in a yet unidentified mechanism, in phagocytic cells and DCs, and thereby may control the levels of IL12p70 production by this mechanism. Enhanced IL12 release observed in ERAP1-KO mice upon stimulation (rEA, Ad) is likely responsible for augmented activation of NK and NKT cells (see Discussion).

## Discussion

It has been well demonstrated that ERAP1 plays a critical role during antigen presentation, shaping the repertoire of antigenic peptides presented on MHC class I molecules, and thereby mediating adaptive immune responses to pathogens, such as bacteria and viruses [2], [6], [40]. In addition to peptide trimming and antigen presentation, ERAP1 has been shown to be involved in the cleavage of several cytokine cell surface receptors indirectly via enhancing the activity of, yet-unknown, metallopeptidases [41]. However, the mechanisms underlying potential ERAP1 involvement in innate immune responses, as well the full extent of this involvement, are not well understood. In this study, we have now found that the presence of a functional ERAP1 protein appears necessary to regulate several important aspects of the innate immune system, and can be evidenced during responses to several forms of inflammatory stimuli.

For example, upon exposure to specific inflammatory stimuli, lack of ERAP1 can result in enhanced gene expression (and production) of multiple cytokines and chemokines, pathogen recognition receptors, and an increased functional capacity of DCs and macrophages to carry out phagocytosis, both *in vitro* and *in vivo*. Lack of ERAP1 also appears to alter the proportions of mature NK cell populations present in mice, with a lack of ERAP1 activity resulting in the presence of higher levels of Stage F mNK cells, and significantly lower levels of Stage E mNK cells in their population of splenocytes, as compared to splenocytes derived from WT mice. In addition, we have found that ERAP1 is involved in the activation of NK and NKT cells, as well in suppressing excessive inductions/proliferation of Stage F mNK cells shortly after exposure to pro-inflammatory stimuli. Examination of these responses using rEA, a potent stimulator of mouse [18], [25] and human NK cells [42] revealed that ERAP1 may act to suppress licensing of NK cells, and that the presence of increased numbers of licensed NK cells may be responsible for the enhanced innate immune responses noted in ERAP1-KO mice exposed to pro-inflammatory stimuli such as Ad viruses and rEA.

Other studies have indirectly suggested influences of ERAP1 on very specific sub-elements of the innate immune system (i.e.: a role in membrane shedding of cytokine receptors (IL6Rα, TNFRI)) *in vitro,* however, similar roles *in vivo* were not confirmed [12], [13], [14]. Recently, the lack of ERAP1 expression in tumor cells was suggested to correlate with an enhanced ability of NK cells to efficiently kill ERAP1 deficient tumor cells, possibly due to lack of regulatory MHC class I/Ly49C interactions [16], [17]. Together, the findings suggest a more global role of the ERAP1 protein in modulating the innate immune system, as well innate immune responses during the earliest stages of pathogen recognition.

The mechanisms underlying this capability are still somewhat unclear. It has been previously confirmed that complete lack of ERAP1 protein during embryonic development does not alter the total number of B cells, CD4^+^, and CD8^+^ T cells in the spleens, lymph nodes, and thymuses of adult C57BL/6 mice, suggesting that lack of ERAP1 does not impact upon the function of these cell lineages due to quantitative deficiencies [9]. This indirectly supports the notion that ERAP1 may have an important direct role in modulating innate immune cell functions due to its specific impact on NK cell maturational status and licensing, and these roles may predispose to alterations in innate immune responses to pro-inflammatory stimuli generally.

Expression of several transcription factors, as well as signaling by NK cell surface receptors have also been shown to regulate NK cell maturational processes. For example, T-bet, GATA-3, IRF-2, and IL15Rα have been shown to play important roles in NK maturation [33], [43]. In our studies, we found that the base-line expression levels of IL15 were significantly increased in the tissues of ERAP1-KO mice as compared to WT mice. It is possible that in response to pro-inflammatory stimuli, the enhanced activation of NK cells, the improved production of IFNγ, and the increased frequency of stage F matured NK cells in ERAP1-KO mice is primarily due to the presence of increased levels of IL15, (possibly by increasing IL15Rα signaling within NK cells) however, future studies beyond the scope of this report will be required to test this interesting hypothesis.

How can ERAP1 be involved in such diverse aspects of the host innate immune response? There are several explanations that can be considered when attempting to address this question. Several aspects of ERAP1 regulation of the innate immune system may yet reside in ERAP1’s ability to modulate loading of peptides onto the MHC class I molecule. For example, NK cells express a variety of inhibitory receptors that regulate their overall levels of activation [44]. One well-documented NK inhibitory mechanism in mice is dependent upon the interaction between the Ly49 NK inhibitory receptor and sufficient amounts of peptide loaded MHC class I molecules being present on the cell surface [45]. We and others have shown that loss of ERAP1 protein results in reduced MHC class I expression, due to lack of availability of properly trimmed peptides in the endoplasmic reticulum [16], [17], [46], [47]. Lack of proper cell surface levels of MHC class I would result in reduced Ly49-mediated inhibitory signals within NK cells during innate immune system development and stimulation and could result in altered frequencies of later Stage NK cells, as well their heightened activation during an inflammatory event [40]. The fact that ERAP1-KO mice express and produce excessive amounts of soluble factors such as IL12 and type I IFNs in response to inflammatory stimuli (known potent activators of NK cells and inducers of IFNγ secretion) may further exacerbate excessive NK cell activation in ERAP1-KO mice exposed to pro-inflammatory stimuli [48], [49].

The activation of NKT cells is not only dependent upon IL12 and type I IFNs, but also other signals, such as co-stimulation provided by enhanced expression of surface receptors (i.e.: mCD40 and OX40) on antigen presenting cells [50]. Enhanced activation of NKT cells can also promote the initial activation of DCs and/or NK cells, thereby further augmenting DC- and NK-dependent innate immune responses [51]. Finally, NKT cells have been shown to inhibit the function of myeloid-derived suppressor cells (MDSCs), which suppress immune responses with nitric oxide synthase 2 (NOS2) and arginase 1 [52]. It is possible that the exaggerated innate immune responses generated in ERAP1-KO mice exposed to inflammatory agents is regulated by a mechanism that involves NKT cell-mediated inhibition of MDSCs.

Our studies also found that the phagocytic activity of macrophages and dendritic cells were dramatically enhanced in response to innate stimuli in ERAP1-KO mice, as compared to WT mice. The process of phagocytosis is a major early functional response of the innate immune system, acting as an initial barrier against the spread of invading pathogens. Phagocytic activity can be induced by a number of signals, including activation of the complement system (C3 cleavage in particular [53]), or the presence of elevated levels of small proteins known as defensins. In addition, over-expression of TLRs and other PRRs on the surface of phagocytic cells, or activation of endothelial cells and expression of adhesion molecules, such as ICAM1 or e-Selectin, are also signals known to promote efficient localization of phagocytes to the site of infection/tissue damage [39], [54], [55]. We have found that in response to inflammatory stimuli, ERAP1 deficient mice exhibit exaggerated levels of transcriptional activation of several PRRs (including TLRs), ICAM1, e-Selectin, and type I IFNs. Enhanced phagocytic activity is also known to trigger increased IL12 production; however, IL12 release itself promotes enhanced phagocytosis by DCs and macrophages [38], [56]. Together with our finding that the basal levels of TNFα gene transcription were also found to be increased in ERAP1-KO mice, these factors may all potentially contribute to an increased macrophage phagocytic activity of ERAP1-KO mice.

Overstimulation of the innate immune system and signaling pathways has also been shown to promote acute and chronic inflammatory states, and may therefore aid the progression of autoimmune diseases [57], [58]. Several genome-wide association studies (GWAS) have documented a critical role for ERAP1, with single nucleotide polymorphisms (SNPs) in ERAP1 being associated with predisposing individuals to autoimmune diseases [59], [60], [61]. For example, the presence of specific ERAP1 SNPs have correlated with the presence of several pathological conditions such as the chronic inflammatory disorder ankylosing spondylitis (AS) [62], [63], type I diabetes (T1D) [64], multiple sclerosis (MS), Crohn’s disease (CD) [65], Behcet's disease [66] and hypertension [67]. Currently, the presumed association of ERAP1 and autoimmune diseases has been suggested to be primarily mediated by its known role in MHC class I antigen presentation pathways, and potential downstream effects on T- and B-cell adaptive immune responses. However, our results suggest that a non-MHC role of ERAP1, that involves initial innate immune system modulation, as well responses to pro-inflammatory stimuli may also be playing a role in these autoimmune disease associations. It may be that the presence of disease promoting ERAP1 SNPs may significantly influence ERAP1’s ability to modulate key aspects of the innate immune system during early pathogen recognition or during exposure to self peptides during an inflammatory event. This lack of regulation of the innate immune system by fully functional ERAP1 proteins may result in enhancing the likelihood of downstream precipitation of autoimmune diseases such as ankylosing spondylitis. Furthermore, NK cells have been shown to be involved in several autoimmune diseases directly or indirectly through their interaction with DCs, macrophages or T cells, thereby inducing exaggerated inflammation or favoring the adaptive T and/or B cells autoimmune responses [68], [69]. Thus, the association of ERAP1 and autoimmune diseases may be mediated by its role in regulating NK cell development, maturation, as well NK roles in acute innate immune responses. Future studies addressing these hypotheses in the respective disease populations will be required to verify these notions.

## Supporting Information

Figure S1 **Mice lacking ERAP1 exhibit increased activation of NKT cells in the spleen in response to rEA stimuli.** C57BL/6 WT and ERAP1-KO mice were either mock (PBS) injected or intraperitoneally injected with 100 ng/mouse of rEA protein. Splenocytes were prepared at 6 hpi, processed, stained for expression of surface markers (intracellular staining was performed for IFNγ), and FACS sorted as described in Materials and Methods. **(A)** CD69 activation NK (top) cells and by NKT (bottom) cells, **(B)** IFNγ release by NK (top) cells and by NKT (bottom) cells are shown. **(C)** CD69 activation of splenic NK cells at 12hpi. Bars represent mean ± SEM. Representative plots are shown. Statistical analysis was completed using a one-way ANOVA with a Student-Newman-Keuls post-hoc test. n = 4–7 for all groups of mice. **, *** - indicate values, statistically different from those in mock-injected mice, p<0.001, p<0.0001, respectively.
(TIFF)

Figure S2 **Mice lacking ERAP1 exhibit significantly augmented activation of NKT cells in response to Adenovirus stimuli.** C57BL/6 WT and ERAP1-KO mice were either mock (PBS) injected or intramuscularly injected with 2×10^10^ vp/mouse of Ad-HIV-Gag. Splenocytes were harvested at 12 hpi, processed, stained for expression of surface markers (intracellular staining was performed for IFNγ), and analyzed by FACS as described in Materials and Methods. Activation and IFNγ release by **(A)** NKT cells and **(B)** NK cells is shown. Bars represent mean ± SEM. Representative plots are shown. Statistical analysis was completed using a one-way ANOVA with a Student-Newman-Keuls post-hoc test. n = 4 for all mock-injected groups, n = 6 for all Ad-injected groups. *, ** - indicate values, statistically different from those in mock-injected mice, p<0.05, p<0.001, respectively.
(TIFF)

Figure S3 **Mice lacking ERAP1 exhibit similar activation of B cells, CD8^+^ and CD8^−^ T cells in response to Adenovirus stimuli.** C57BL/6 WT and ERAP1-KO mice were either mock (PBS) injected or intramuscularly injected with 2×10^10^ vp/mouse of Ad-HIV-Gag. Splenocytes were harvested at 12 hpi, processed, stained for expression of surface markers, and analyzed by FACS as described in Materials and Methods. CD69 activation of **(A)** B cells, **(B)** CD8^+^ CD3^+^ T cells and **(C)** CD8**^−^** CD3^+^ T cells are shown. Bars represent mean ± SEM. Representative plots are shown. Statistical analysis was completed using a one-way ANOVA with a Student-Newman-Keuls post-hoc test. n = 4 for all mock-injected groups, n = 6 for all Ad-injected groups. *, ** - indicate values, statistically different from those in mock-injected mice, p<0.05, p<0.001, respectively.
(TIFF)

Figure S4 **ERAP1-KO and WT mice express similar number of mature NK cells.** WT C57BL/6 and ERAP1-KO mice (n = 4 for mock and n = 7 for rEA injected groups of mice) were either mock (PBS) injected or intraperitoneally injected with 100 ng/mouse of rEA protein. Splenocytes were harvested at 12 hpi, processed, stained for expression of MK1.1 and DX5 on cells (gated on CD3− cells), and FACS analysis was performed as described in materials and methods. Numbers indicate mature NK cells (NK1.1+DX5+) in spleens of ERAP1-KO or WT mice. The bars represent mean ± SEM. Statistical analysis was completed using a one-way ANOVA with a Student-Newman-Keuls post-hoc test. p<0.05 was deemed a statistically significant difference. *** - indicate values, statistically different from those in mock-injected mice, p<0.001.
(TIFF)

Figure S5 **NK cells of ERAP1-KO mice are licensed.** WT C57BL/6 and ERAP1-KO mice (n = 4 for mock and n = 7 for rEA injected groups of mice) were either mock (PBS) injected or intraperitoneally injected with 100 ng/mouse of rEA protein. Splenocytes were harvested at 12 hpi, processed, stained for expression of Ly49C and production of IFNγ from NK cells (gated on CD3− cells) following stimulation with the anti-NK1.1 or IL2+IL18 cytokines, as described in materials and methods. (A) Representative figures of FACS analysis for IFNγ production from anti-NK1.1 or IL2+IL18 stimulated splenocytes derived from naive WT, ERAP1-KO, or β2m-KO mice. (B) Frequency of IFNγ-producing Ly49C+ NK1.1+CD3− cells derived from WT, ERAP1-KO, or β2m-KO mice following stimulation with 0.6 or 2 µg of anti-NK1.1. (C) Frequency of IFNγ-producing Ly49C- NK1.1+CD3− cells derived from WT, ERAP1-KO, or β2m-KO mice following stimulation with 0.6 or 2 µg of anti-NK1.1. (D) Frequency of IFNγ-producing Ly49C+ (left) and Ly49C- (right) NK1.1+CD3− cells derived from WT, ERAP1-KO, or β2m-KO mice following stimulation with IL2 and IL18 cytokines. The bars represent mean ± SEM.
(TIFF)

Figure S6 **Increased expression of Ly49C on NK cells of ERAP1-KO mice.** Splenocytes derived from naïve WT, ERAP1-KO, and β2m-KO mice (n = 2) were stained for the expression of Ly49C and Ly49A on CD3−NK1.1+ NK cells. Representative figure of FACS analysis is shown.
(TIFF)

Figure S7 **Adenovirus vectors transduce C57BL/6 WT and ERAP1-KO mice with similar efficacy. (A)** qPCR based quantification of Ad5-HIV-Gag genomes in livers harvested from C57BL/6 WT or ERAP1-KO mice at 6 hpi was performed as described in Materials and Methods. The bars represent Mean ± SD. Statistical analysis was completed using two-tailed Student t-test to compare 2 groups of virus injected animals. No significant differences were found. **(B)** C57BL/6 WT and ERAP1-KO mice were either mock (PBS) injected or intramuscularly injected with 2×10^10^ vp/mouse of Ad-GFP. Splenocytes were collected at 12 hpi, processed and either analyzed for GFP expression, FITC channel, total lymphocytes, or stained for expression of CD3 and NK.1.1 surface markers, and analyzed by FACS as described in Materials and Methods in order to study Ad transduction capabilities in **(C)** NK cells. Bars represent mean ± SEM. Representative plots are shown. Statistical analysis was completed using a one-way ANOVA with a Student-Newman-Keuls post-hoc test. n = 4 for all groups of mice. * - indicate values, statistically different from those in mock-injected mice, p<0.05.
(TIFF)
