# Supplementary figures and images for: Endoplasmic Reticulum Aminopeptidase-1 Functions Regulate Key Aspects of the Innate Immune Response
Source: PLoS One. 2013 Jul 24;8(7):e69539. doi: 10.1371/journal.pone.0069539 (PMC3722114; doi:10.1371/journal.pone.0069539)

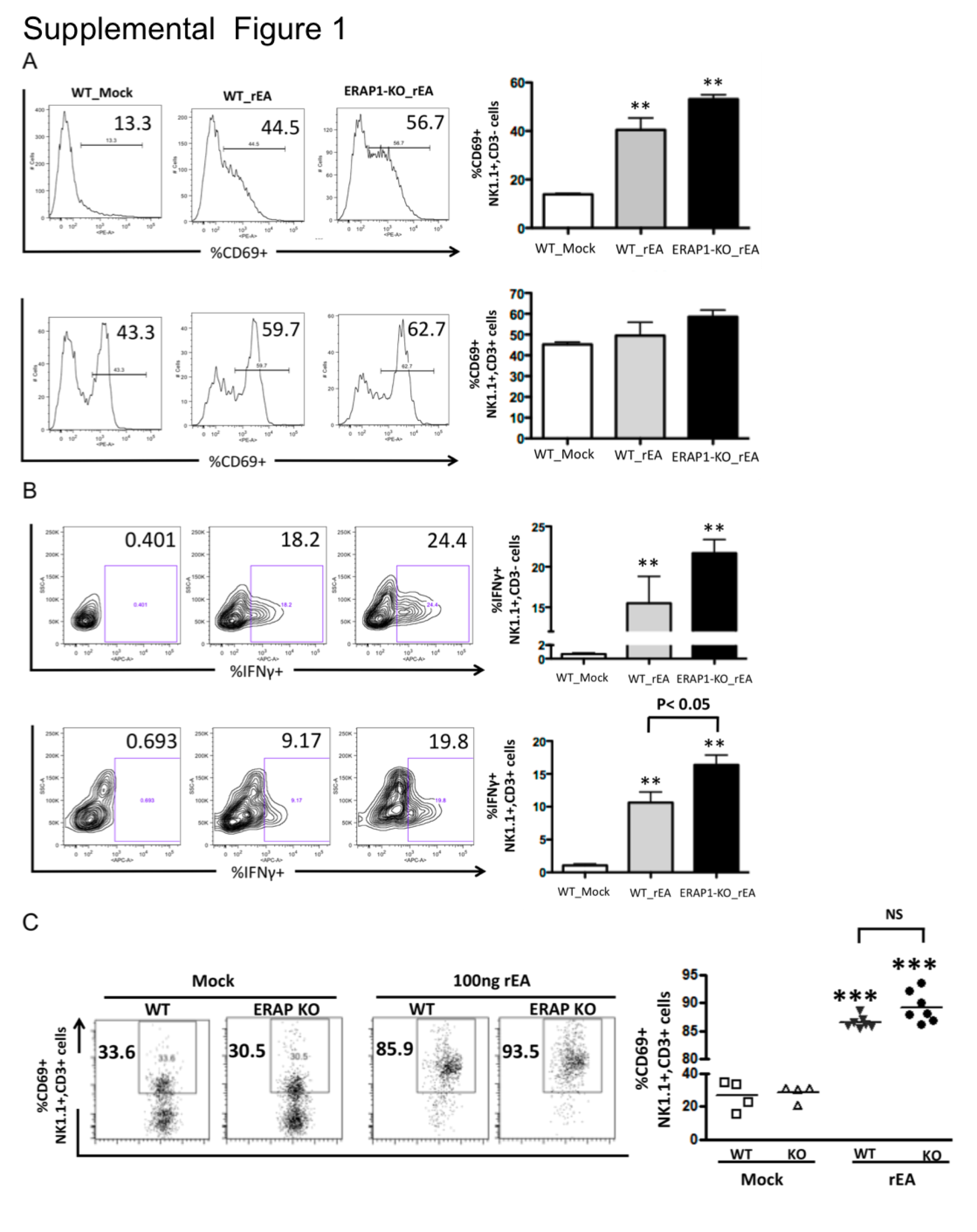

Supplement: Figure S1 — Mice lacking ERAP1 exhibit increased activation of NKT cells in the spleen in response to rEA stimuli. C57BL/6 WT and ERAP1-KO mice were either mock (PBS) injected or intraperitoneally injected with 100 ng/mouse of rEA protein. Splenocytes were prepared at 6 hpi, processed, stained for expression of surface markers (intracellular staining was performed for IFNγ), and FACS sorted as described in Materials and Methods. (A) CD69 activation NK (top) cells and by NKT (bottom) cells, (B) IFNγ release by NK (top) cells and by NKT (bottom) cells are shown. (C) CD69 activation of splenic NK cells at 12hpi. Bars represent mean ± SEM. Representative plots are shown. Statistical analysis was completed using a one-way ANOVA with a Student-Newman-Keuls post-hoc test. n = 4–7 for all groups of mice. **, *** - indicate values, statistically different from those in mock-injected mice, p<0.001, p<0.0001, respectively. (TIFF) [file pone.0069539.s001.tiff]

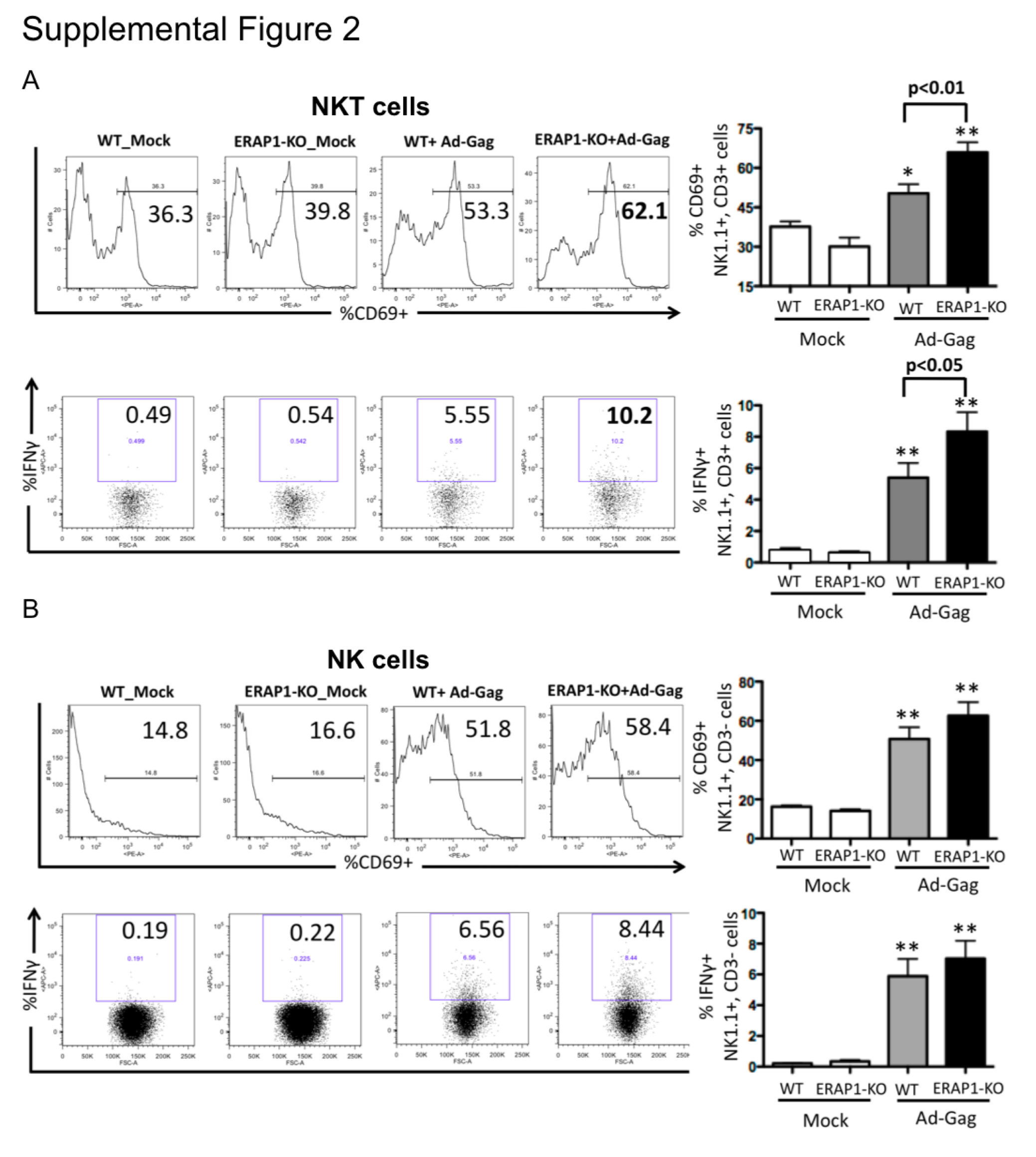

Supplement: Figure S2 — Mice lacking ERAP1 exhibit significantly augmented activation of NKT cells in response to Adenovirus stimuli. C57BL/6 WT and ERAP1-KO mice were either mock (PBS) injected or intramuscularly injected with 2×10^10^ vp/mouse of Ad-HIV-Gag. Splenocytes were harvested at 12 hpi, processed, stained for expression of surface markers (intracellular staining was performed for IFNγ), and analyzed by FACS as described in Materials and Methods. Activation and IFNγ release by (A) NKT cells and (B) NK cells is shown. Bars represent mean ± SEM. Representative plots are shown. Statistical analysis was completed using a one-way ANOVA with a Student-Newman-Keuls post-hoc test. n = 4 for all mock-injected groups, n = 6 for all Ad-injected groups. *, ** - indicate values, statistically different from those in mock-injected mice, p<0.05, p<0.001, respectively. (TIFF) [file pone.0069539.s002.tiff]

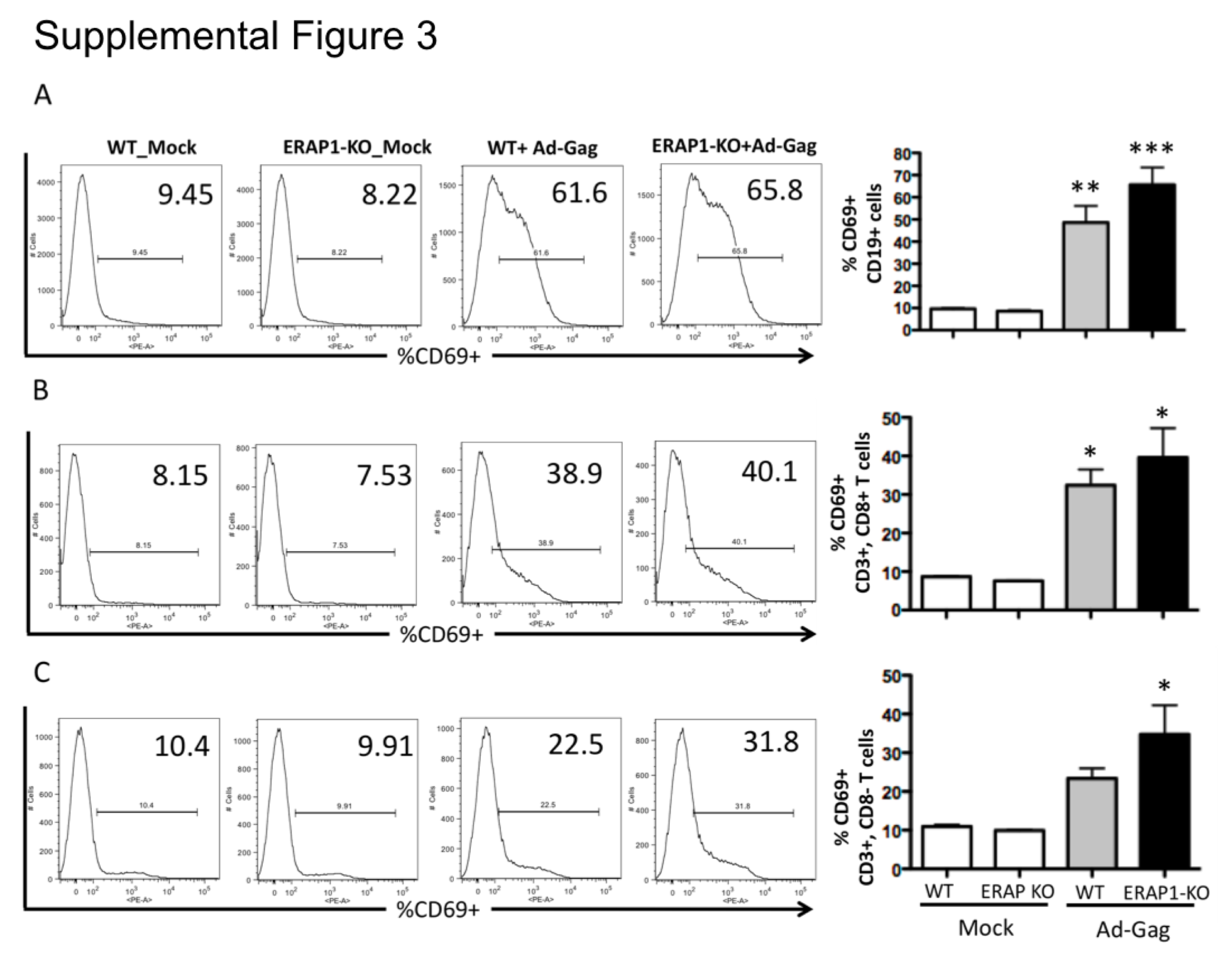

Supplement: Figure S3 — Mice lacking ERAP1 exhibit similar activation of B cells, CD8+ and CD8− T cells in response to Adenovirus stimuli. C57BL/6 WT and ERAP1-KO mice were either mock (PBS) injected or intramuscularly injected with 2×10^10^ vp/mouse of Ad-HIV-Gag. Splenocytes were harvested at 12 hpi, processed, stained for expression of surface markers, and analyzed by FACS as described in Materials and Methods. CD69 activation of (A) B cells, (B) CD8+ CD3+ T cells and (C) CD8− CD3+ T cells are shown. Bars represent mean ± SEM. Representative plots are shown. Statistical analysis was completed using a one-way ANOVA with a Student-Newman-Keuls post-hoc test. n = 4 for all mock-injected groups, n = 6 for all Ad-injected groups. *, ** - indicate values, statistically different from those in mock-injected mice, p<0.05, p<0.001, respectively. (TIFF) [file pone.0069539.s003.tiff]

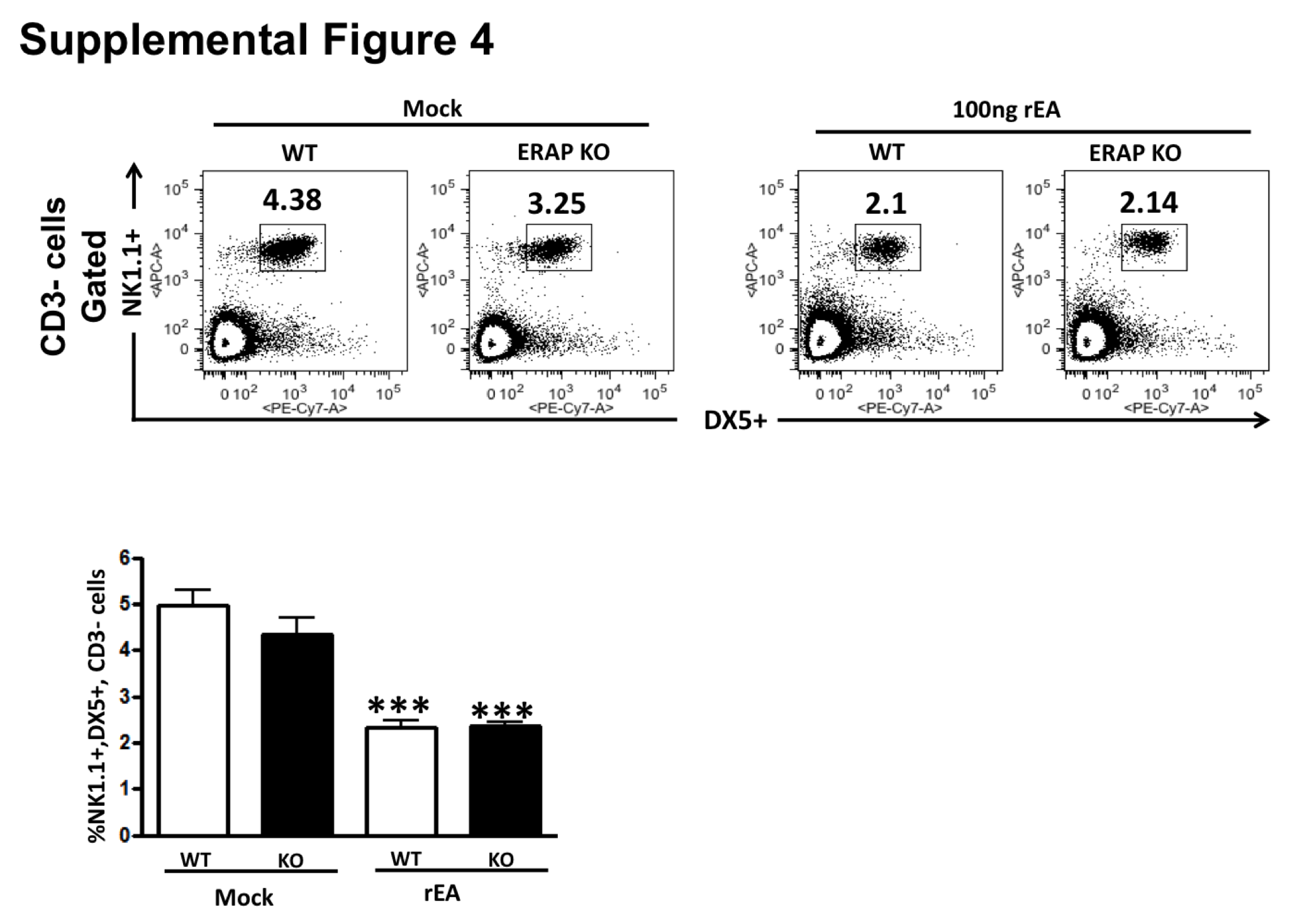

Supplement: Figure S4 — ERAP1-KO and WT mice express similar number of mature NK cells. WT C57BL/6 and ERAP1-KO mice (n = 4 for mock and n = 7 for rEA injected groups of mice) were either mock (PBS) injected or intraperitoneally injected with 100 ng/mouse of rEA protein. Splenocytes were harvested at 12 hpi, processed, stained for expression of MK1.1 and DX5 on cells (gated on CD3− cells), and FACS analysis was performed as described in materials and methods. Numbers indicate mature NK cells (NK1.1+DX5+) in spleens of ERAP1-KO or WT mice. The bars represent mean ± SEM. Statistical analysis was completed using a one-way ANOVA with a Student-Newman-Keuls post-hoc test. p<0.05 was deemed a statistically significant difference. *** - indicate values, statistically different from those in mock-injected mice, p<0.001. (TIFF) [file pone.0069539.s004.tiff]

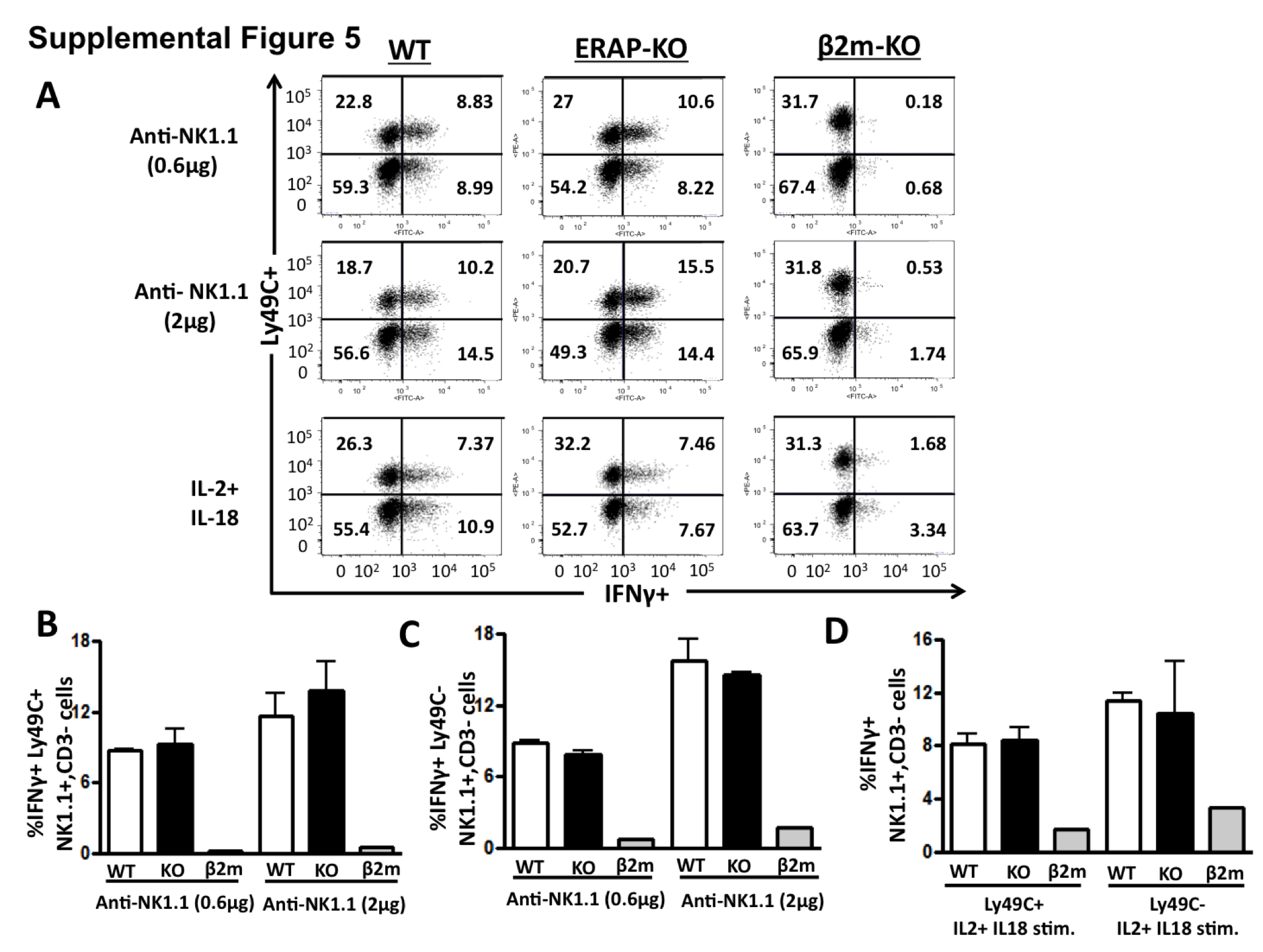

Supplement: Figure S5 — NK cells of ERAP1-KO mice are licensed. WT C57BL/6 and ERAP1-KO mice (n = 4 for mock and n = 7 for rEA injected groups of mice) were either mock (PBS) injected or intraperitoneally injected with 100 ng/mouse of rEA protein. Splenocytes were harvested at 12 hpi, processed, stained for expression of Ly49C and production of IFNγ from NK cells (gated on CD3− cells) following stimulation with the anti-NK1.1 or IL2+IL18 cytokines, as described in materials and methods. (A) Representative figures of FACS analysis for IFNγ production from anti-NK1.1 or IL2+IL18 stimulated splenocytes derived from naive WT, ERAP1-KO, or β2m-KO mice. (B) Frequency of IFNγ-producing Ly49C+ NK1.1+CD3− cells derived from WT, ERAP1-KO, or β2m-KO mice following stimulation with 0.6 or 2 µg of anti-NK1.1. (C) Frequency of IFNγ-producing Ly49C- NK1.1+CD3− cells derived from WT, ERAP1-KO, or β2m-KO mice following stimulation with 0.6 or 2 µg of anti-NK1.1. (D) Frequency of IFNγ-producing Ly49C+ (left) and Ly49C- (right) NK1.1+CD3− cells derived from WT, ERAP1-KO, or β2m-KO mice following stimulation with IL2 and IL18 cytokines. The bars represent mean ± SEM. (TIFF) [file pone.0069539.s005.tiff]

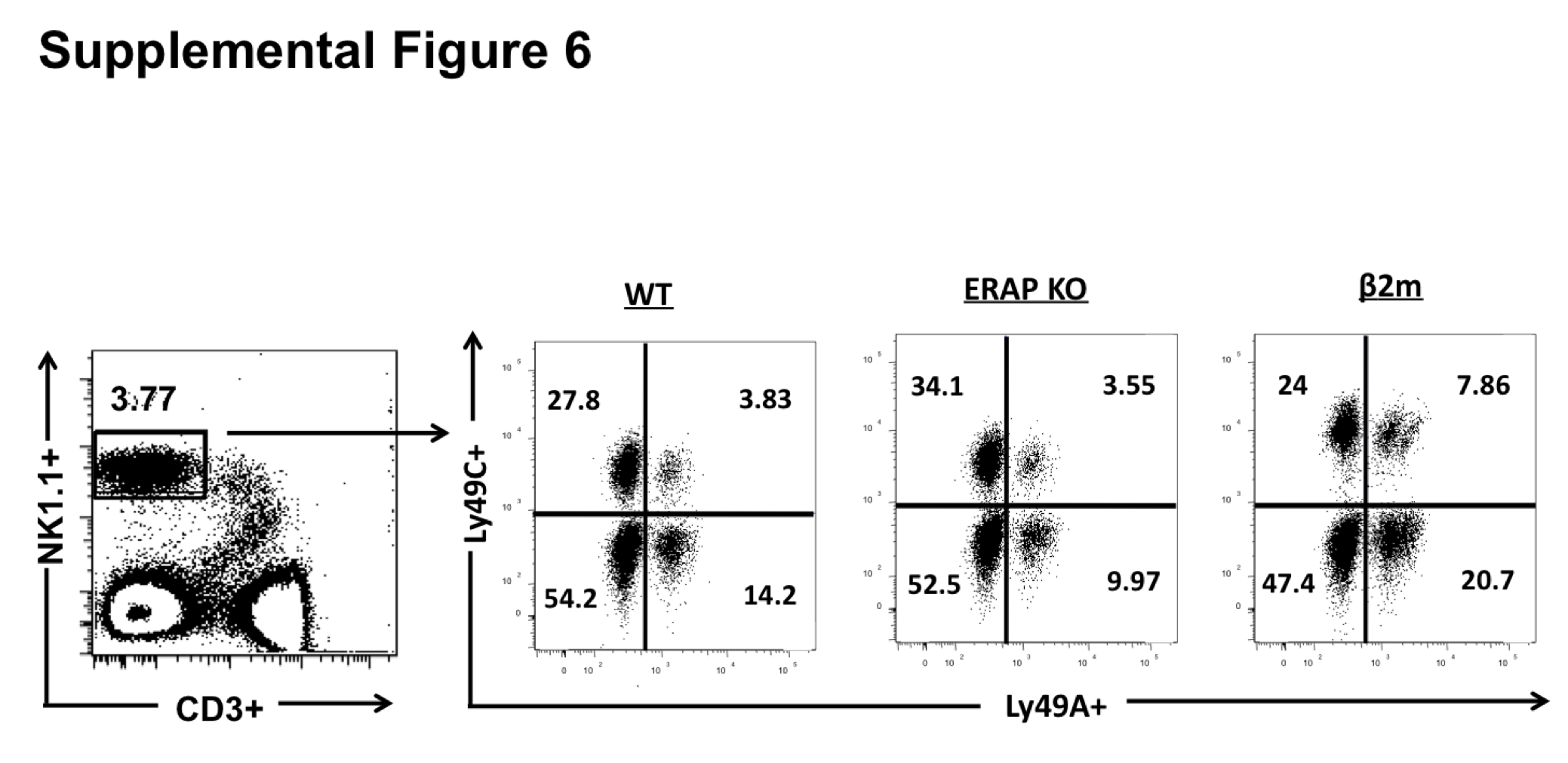

Supplement: Figure S6 — Increased expression of Ly49C on NK cells of ERAP1-KO mice. Splenocytes derived from naïve WT, ERAP1-KO, and β2m-KO mice (n = 2) were stained for the expression of Ly49C and Ly49A on CD3−NK1.1+ NK cells. Representative figure of FACS analysis is shown. (TIFF) [file pone.0069539.s006.tiff]

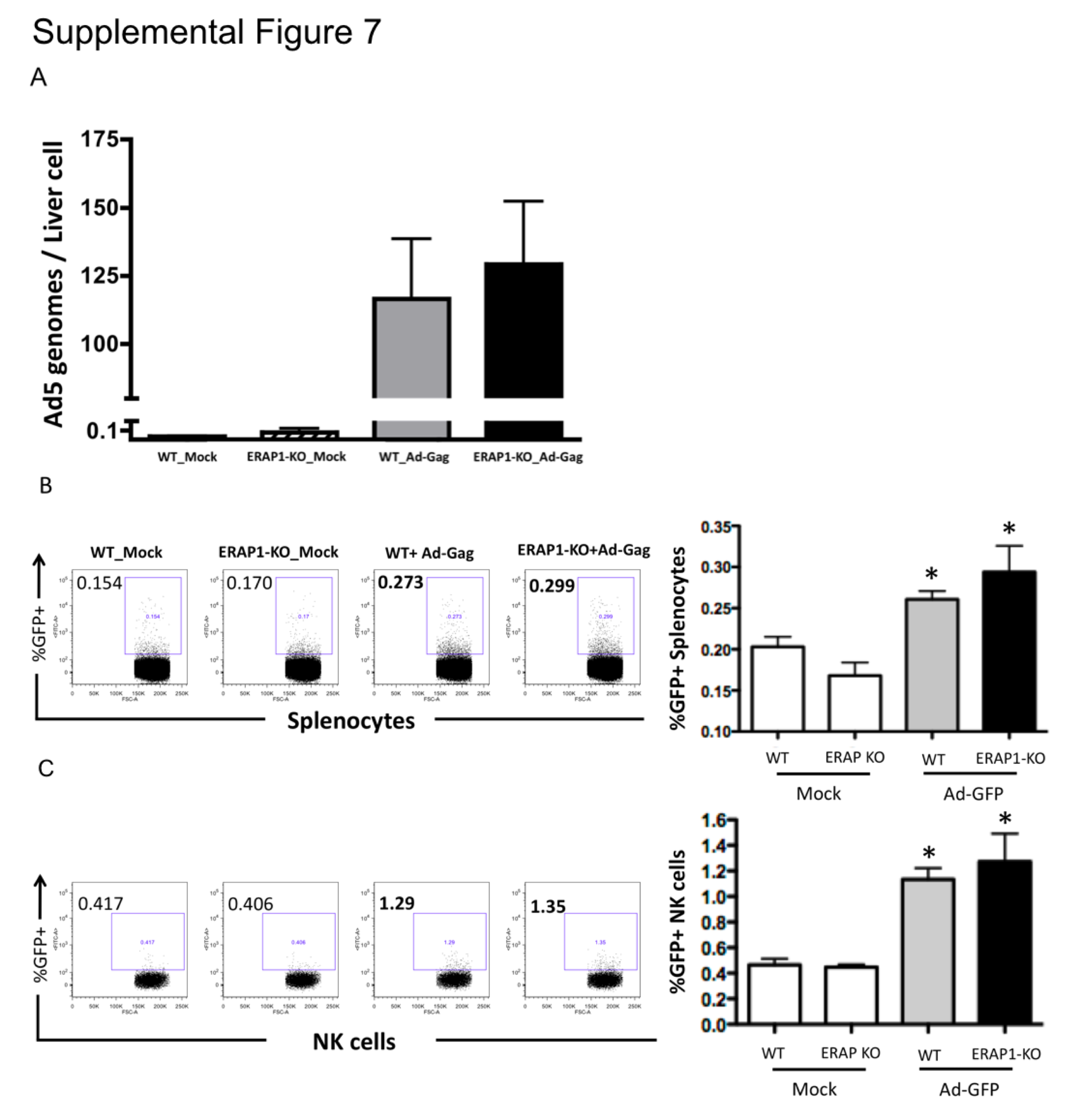

Supplement: Figure S7 — Adenovirus vectors transduce C57BL/6 WT and ERAP1-KO mice with similar efficacy. (A) qPCR based quantification of Ad5-HIV-Gag genomes in livers harvested from C57BL/6 WT or ERAP1-KO mice at 6 hpi was performed as described in Materials and Methods. The bars represent Mean ± SD. Statistical analysis was completed using two-tailed Student t-test to compare 2 groups of virus injected animals. No significant differences were found. (B) C57BL/6 WT and ERAP1-KO mice were either mock (PBS) injected or intramuscularly injected with 2×10^10^ vp/mouse of Ad-GFP. Splenocytes were collected at 12 hpi, processed and either analyzed for GFP expression, FITC channel, total lymphocytes, or stained for expression of CD3 and NK.1.1 surface markers, and analyzed by FACS as described in Materials and Methods in order to study Ad transduction capabilities in (C) NK cells. Bars represent mean ± SEM. Representative plots are shown. Statistical analysis was completed using a one-way ANOVA with a Student-Newman-Keuls post-hoc test. n = 4 for all groups of mice. * - indicate values, statistically different from those in mock-injected mice, p<0.05. (TIFF) [file pone.0069539.s007.tiff]
